# Scaling of Protein Function across the Tree of Life

**DOI:** 10.1093/gbe/evad214

**Published:** 2023-11-26

**Authors:** Riddhi Gondhalekar, Christopher P Kempes, Shawn Erin McGlynn

**Affiliations:** Earth-Life Science Institute, Tokyo Institute of Technology, Tokyo, Japan; School of Life Sciences and Technology, Tokyo Institute of Technology, Tokyo, Japan; The Santa Fe Institute, Santa Fe, New Mexico, USA; Earth-Life Science Institute, Tokyo Institute of Technology, Tokyo, Japan; School of Life Sciences and Technology, Tokyo Institute of Technology, Tokyo, Japan; Blue Marble Space Institute of Science, Seattle, Washington, USA; Center for Sustainable Resource Science, RIKEN, Saitama, Japan

**Keywords:** scaling laws, evolution, genome, proteins, major transitions

## Abstract

Scaling laws are a powerful way to compare genomes because they put all organisms onto a single curve and reveal nontrivial generalities as genomes change in size. The abundance of functional categories across genomes has previously been found to show power law scaling with respect to the total number of functional categories, suggesting that universal constraints shape genomic category abundance. Here, we look across the tree of life to understand how genome evolution may be related to functional scaling. We revisit previous observations of functional genome scaling with an expanded taxonomy by analyzing 3,726 bacterial, 220 archaeal, and 79 unicellular eukaryotic genomes. We find that for some functional classes, scaling is best described by multiple exponents, revealing previously unobserved shifts in scaling as genome-encoded protein annotations increase or decrease. Furthermore, we find that scaling varies between phyletic groups at both the domain and phyla levels and is less universal than previously thought. This variability in functional scaling is not related to taxonomic phylogeny resolved at the phyla level, suggesting that differences in cell plan or physiology outweigh broad patterns of taxonomic evolution. Since genomes are maintained and replicated by the functional proteins encoded by them, these results point to functional degeneracy between taxonomic groups and unique evolutionary trajectories toward these. We also find that individual phyla frequently span scaling exponents of functional classes, revealing that individual clades can move across scaling exponents. Together, our results reveal unique shifts in functions across the tree of life and highlight that as genomes grow or shrink, proteins of various functions may be added or lost.

**Significance**

Life requires a set of functionalities, but the proportionality of these functionalities to one another can vary between organisms. We investigated how functional groups of proteins scale across the tree of life and found variability between phylogenetic groups. Additionally, we found that some functional categories show shifts in their scaling as the total functional category amount increases and that individual phylogenetic groups exist across these shifts. Our work suggests plasticity in functional category scaling across the tree of life, and also that this plasticity is not a phylogenetic variable.

## Introduction

Scaling relationships are ubiquitous and are suggestive that a set of rules governs how a system works (Corominas-Murtra et al. 2018). In biological systems, a common challenge for understanding the history of life, extant species diversity, and the possibilities for life beyond Earth is to find biological laws that systematize biology in these different contexts. As we know it on Earth today, life is unified in using a conserved set of proteins (Harris et al. 2003; Martinez-Gutierrez and Aylward 2021), but organisms differ dramatically in their unique and shared protein complements (e.g., Charlebois and Doolittle 2004; Lapierre and Gogarten 2009). Despite this diversity, a variety of scaling relationships across the tree of life have been identified, suggesting universal constraints shape processes and form across lineages, even if accomplished in evolutionarily independent ways (van Nimwegen 2003; Brown et al. 2004; Savage et al. 2004; Molina and van Nimwegen 2009; DeLong et al. 2010; Grilli et al. 2012; Kempes et al. 2012, 2016, 2017; De Lazzari et al. 2017; West 2017; Gagler et al. 2022). By identifying the connection between scaling relationships and physical constraints, it may be possible to demonstrate that they represent laws. For example, in bacteria, scaling in physiology, metabolism, and growth is often due to connections between physical constraints and cell size (DeLong et al. 2010; Kempes et al. 2012, 2016).

One class of scaling relationships is those observed comparing the total number of annotated proteins to the abundance of specific functional classes. Genome size is a complex trait with relationships to temperature (Sato et al. 2020), physiology (Nielsen et al. 2021), lineage history (Martinez-Gutierrez and Aylward 2022), and cell size (Lynch 2020). When genomes expand or contract, function may be gained or lost in a nonuniform way. Starting with a seminal study by van Nimwegen (2003) and subsequently expanded by others (Konstantinidis and Tiedje 2004; Cordero and Hogeweg 2007; Molina and van Nimwegen 2008; Grilli et al. 2012; De Lazzari et al. 2017; Gagler et al. 2022), the number of genes in a functional category has been found to scale as a power law with the total number of annotated genes in the genome (van Nimwegen 2003; Molina and van Nimwegen 2009).

Such scaling patterns have been demonstrated using functional annotations, which indicated that metabolic and electron transfer processes scale uniquely between clades, highlighting that different metabolisms have different regulatory and energy demands, whereas other genomically encoded processes, such as translation, scale similarly across clades (van Nimwegen 2003; Molina and van Nimwegen 2009; De Lazzari et al. 2017). Recent work has also suggested that at the level of broad enzyme “operations” (E.C. level 1) categories, there are no great shifts across taxa in scaling exponents suggesting a greater universality at the level of enzyme operations than observed in scaling of specific functions (Gagler et al. 2022). This sets up an interesting question about how functional categories scale, as this categorization projects the same enzymes onto more discrete physiological processes captured by homologous groups rather than broad E.C. level 1 categories of enzyme function (Gagler et al. 2022).

With the much larger number of genomes available today, it is worth revisiting functional scaling relationships first observed by van Nimwegen in 2003 across an expanded phylogenetic diversity. As two examples of many, Thaumarchaeota genomes only became available in the 2010s (Pester et al. 2011), after functional protein scaling relationships were first reported, and metabolisms once thought to be rather restricted have now been found more broadly; for example, methane metabolism seems more widespread across the archaeal tree (see Garcia et al. 2022 and references contained therein). Across this increased genomic and physiological diversity, it may also be possible to test the effects of major evolutionary transitions in connection with known shifts in physiological scaling (DeLong et al. 2010; Kempes et al. 2012) and to test trends within taxa with greater statistical power.

Here, we use clusters of orthologous genes (COG) functional annotations to investigate previous observations with an expanded taxonomy. We make use of the evolutionary genealogy of genes: Non-supervised Orthologous Groups (eggNOG) database (Huerta-Cepas et al. 2019) as a starting data set, which covers more taxonomic diversity than has been used in previous studies, and supplement this by annotating other genomes of interest using the eggNOG annotator: including members of the CPR, DPANN, Melainabacteria, Asgard archaea, and some lower eukaryotes. We find a substantial variation of functional scaling between clades and suggest that some major evolutionary transitions may have occurred in conjunction with changes in functional scaling, which would mirror the physiological shifts observed over these transitions.

## Results and Discussion

We discuss scaling between the COG categories, designating linear scaling or isometric growth for scaling exponents near one, sublinear or fractional dilution for exponents less than one, and superlinear or fractional enrichment for scaling exponents more than one. Evolutionarily, linear scaling would mean as the total functional categories in a group or phyla increase, the number of unique proteins increases at a similar rate. While superlinear would mean that a specific category of proteins increases at a higher proportion than other categories as genomes expand, sublinear scaling would still mean an increase in the number of proteins but at a lower proportion than that of the total functional categories increase. It is important to note that our study does not address copy numbers; scaling exponents reflect only unique sequences in the genome.

### Scaling Shifts within the Bacteria and Archaea Domains

Many scaling theories predict asymptotic behavior at the small and large end of a group, where certain properties go to zero or infinity. For example, growth rates and ribosome content all have asymptotes at either the small or large end of bacteria and/or eukaryotes (Kempes et al. 2012, 2016, 2019). These physiological asymptotes appear in bacteria, microbial eukaryotes, mammals, plants, and insects (Kempes et al. 2019) and can be seen as curvature away from the scaling relationships that capture the intermediate cell sizes. Such behavior is typically caused by a particular constraint becoming overly challenging for physiology. This includes maintenance metabolism overcoming total metabolism for the smallest bacteria and largest unicellular eukaryotes, DNA and protein volumes overpacking the smallest bacteria, growth rate requiring more ribosomes than can fit in the cell for large bacteria, the overdampening of the vascular system in small mammals, and hydraulic limits for the largest trees (e.g., see Kempes et al. 2019 for a review). Thus, we should expect curvature in protein function scaling laws as most aspects of bacterial physiology are defined by scaling laws with asymptotes at the boundaries (Kempes et al. 2012, 2016, 2017, 2019).

**Fig. 1.**
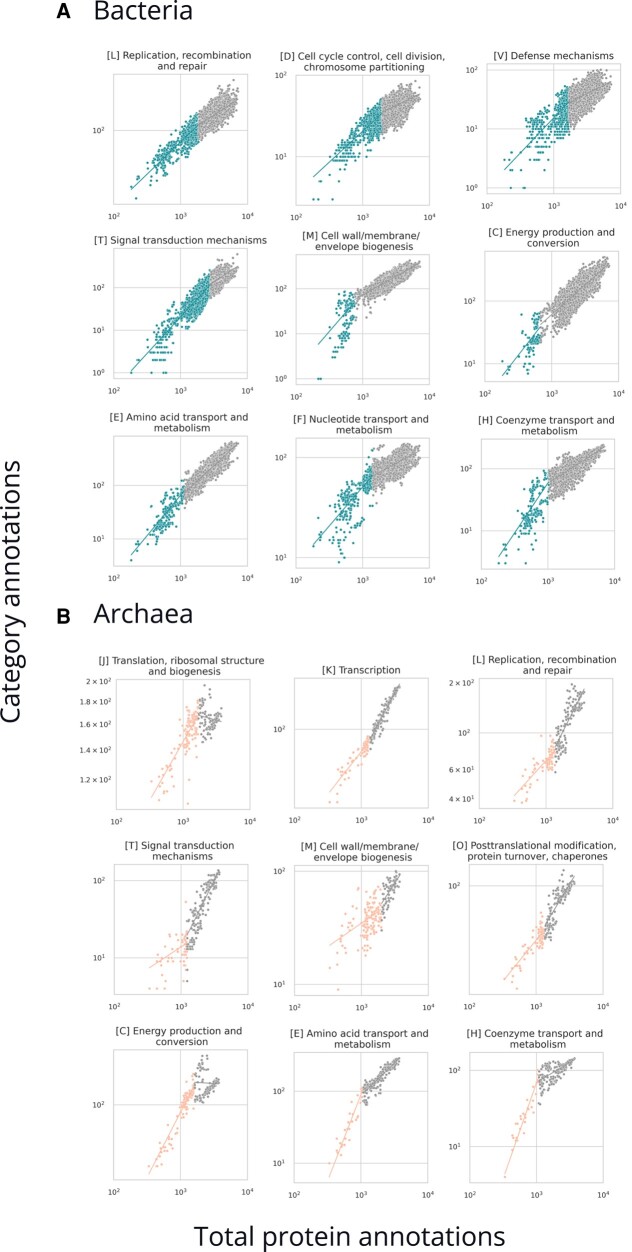
Shifts in functional scaling as observed in (*A*) bacteria and (*B*) archaea shown on breakpoint plots. Axes are in log scale. The *x* axis has total protein annotations (sum of all COG annotations in an organism), and the *y* axis has annotations in a specific category. The plots signify two slopes divided by a breakpoint. Only categories with breakpoints are included.

To search for this in a general manner, we performed a breakpoint analysis to see when there is support for a bilinear fit. In this analysis, we analyzed archaea and bacteria only, because the number of genomes for eukaryotes is relatively small and does not warrant a breakpoint analysis. Plotting the number of COG category annotations against total protein annotations shows that for the case of bacteria and archaea, the best fits for some categories are by more than one line (fig. 1 and table 1). Such scaling shifts may be present in eukaryotes but are not observed in our current work, likely due to the small number of genomes available.

**Table 1:** Domain Level Functional Scaling Slopes with Breakpoints

COG Category	Bacteria	Archaea	Eukaryotes
[A] RNA processing and modification	NA	NA	0.46 ± 0.21
[B] chromatin structure and dynamics	0.41 ± 0.04	0.018 ± 0.219	0.83 ± 0.10
[C] energy production and conversion	Slope 1: 1.34 ± 0.28; slope 2: 0.98 ± 0.02	Slope 1: 1.52 ± 0.09; slope 2: 0.06 ± 0.24	0.82 ± 0.18
[D] cell cycle control, cell division, chromosome partitioning	Slope 1: 0.82 ± 0.03*; slope 2: 0.42 ± 0.03	0.82 ± 0.11*	0.91 ± 0.12*
[E] amino acid transport and metabolism	Slope 1: 1.53 ± 0.14; slope 2: 1.10 ± 0.02	Slope 1: 2.12 ± 0.32; slope 2: 0.91 ± 0.06	0.80 ± 0.18
[F] nucleotide transport and metabolism	Slope 1: 0.81 ± 0.06; slope 2: 0.34 ± 0.01	0.56 ± 0.04	0.83 ± 0.10
[G] carbohydrate transport and metabolism	1.10 ± 0.02	0.79 ± 0.09	1.09 ± 0.13
[H] coenzyme transport and metabolism	Slope 1: 1.64 ± 0.23; slope 2: 0.75 ± 0.02	Slope 1: 2.19 ± 0.31; slope 2: 0.56 ± 0.09	0.68 ± 0.16
[I] lipid transport and metabolism	1.17 ± 0.02*	1.25 ± 0.12*	1.18 ± 0.14*
[J] translation, ribosomal structure, and biogenesis	0.27 ± 0.00	Slope 1: 0.27 ± 0.04; slope 2: −0.04 ± 0.06	0.68 ± 0.10
[K] transcription	1.01 ± 0.01	Slope 1: 0.88 ± 0.09; slope 2: 1.28 ± 0.06	0.74 ± 0.19
[L] replication, recombination, and repair	Slope 1: 0.72 ± 0.03; slope 2: 0.48 ± 0.02	Slope 1: 0.45 ± 0.08; slope 2: 0.87 ± 0.09	0.92 ± 0.16
[M] cell wall/membrane/envelope biogenesis	Slope 1: 2.82 ± 0.51; slope 2: 0.87 ± 0.02	Slope 1: 0.50 ± 0.13; slope 2: 1.17 ± 0.24	1.38 ± 0.23
[N] cell motility	1.39 ± 0.05	0.97 ± 0.20	2.23 ± 0.42
[O] posttranslational modification, protein turnover, chaperones	0.88 ± 0.01	Slope 1: 0.67 ± 0.09; slope 2: 0.97 ± 0.10	1.18 ± 0.10
[P] inorganic ion transport and metabolism	1.18 ± 0.01	1.27 ± 0.05	1.08 ± 0.13
[Q] secondary metabolites biosynthesis, transport, and catabolism	1.72 ± 0.02	1.62 ± 0.11	1.24 ± 0.22
[R] general function prediction only	NA	NA	NA
[S] function unknown	1.32 ± 0.01	1.52 ± 0.06	0.91 ± 0.19
[T] signal transduction mechanisms	Slope 1: 1.72 ± 0.04; slope 2: 1.11 ± 0.07	Slope 1: 0.64 ± 0.30; slope 2: 2.08 ± 0.17	1.60 ± 0.18
[U] intracellular trafficking, secretion, and vesicular transport	0.87 ± 0.02	0.59 ± 0.12	0.71 ± 0.14
[V] defense mechanisms	Slope 1: 1.17 ± 0.08; slope 2: 0.62 ± 0.034	0.78 ± 0.12	1.61 ± 0.20
[W] extracellular structures	NA	0.50 ± 1.16	1.14 ± 0.44
[X] mobilome: prophages, transposons	NA	NA	NA
[Y] nuclear structure	NA	NA	−0.28 ± 0.43
[Z] cytoskeleton	NA	NA	1.45 ± 0.17

Note.—Slopes 1 and 2 indicate categories with breakpoints. Supplementary table S2, Supplementary Material online has Asgard archaea and DPANN and CPR separated from archaea and bacteria, respectively. Exponents with asterisks are within the error bars of each other across all domains.

Scaling shifts can be unique between the archaea and bacteria (fig. 1 and table 1). The categories [L] replication, [T] signal transduction mechanism, [M] cell wall/membrane/envelope biogenesis, [C] energy production and conversion, [E] amino acid transport and metabolism, and [H] coenzyme transport and metabolism are found to be similar in both bacteria and archaea, while [J] translation, [K] transcription, and [O] are unique to the archaea. All scaling shifts in bacteria are all toward less positive exponents as the total number of annotations increases; however, in archaea, most shifts are to higher exponents except for [C], [E], and [H], which decrease as with bacteria. Categories [D] cell cycle control, [V] defense mechanisms, and [F] nucleotide transport and metabolism are unique to displaying scaling shifts with bacteria. These broad differences in the scaling of protein function between archaea and bacteria are surprising since they imply that genome growth is accommodated by functionally distinct processes in the two domains, and the tendency of one category to be enriched or depleted is not necessarily a constant.

### Domain Level View of Functional Scaling

One of the main interests in biological scaling is the universality of scaling relationships, which may indicate shared fundamental constraints, where previously observed patterns in enzyme scaling appear to illustrate universal behavior (Gagler et al. 2022) while physiological scaling tends to illustrate shifts across the major evolutionary transitions (DeLong et al. 2010; Kempes et al. 2012, 2016, 2019). Here, we find that only a few categories share similar exponents across most of the prokaryotes and eukaryotes ([G], [I], [P], [T], and [U]). However, most of these have differences in the normalization constant (*y* intercept) of the scaling across prokaryotes and eukaryotes, and it is only really signal transduction [T] and lipid transport and metabolism [I] that could be argued to follow a single relationship across all unicellular organisms.

Recognizing that some functional categories are best fit by two scaling slopes, we next sought to gain an overview of functional scaling between the archaeal, bacterial, and eukaryotic domains as it occurs between different protein functional classes, looking at the broad groups of the clusters of orthologous groups.

#### Information Storage and Processing: COG Categories A, B, J, K, and L

Ribosomes have been under strong selection pressures and are vital in differentiating the three domains of life. Interestingly, specific ribosomal proteins have been progressively accumulated and/or lost through evolution in the three domains (Lecompte 2002). As reported in previous scaling studies (van Nimwegen 2003; Molina and van Nimwegen 2009; Grilli et al. 2012; Sela et al. 2019), we observed sublinear scaling for category [J] translation, ribosomal structure, and biogenesis across all three domains. Archaea and bacteria both are sublinear, but archaea show an extreme reductive evolutionary scenario with their second slope in figure 1 being near zero (table 1), and have been shown to preserve ribosomal proteins differently than bacteria and eukaryotes, losing up to ten ribosomal proteins in contrast to only four being lost in bacteria and eukaryotes through evolution (Lecompte 2002). Eukaryotes scale with a mildly sublinear scaling exponent of 0.68 ± 0.10. The normalization constants for this category are exceptionally high for all the domains, which indicates large quantities of proteins in this category from the onset of genome evolution as we see it and explains ribosomal proteins involved in several extraribosomal functions, mainly in transcription and regulation (supplementary tables S7 and S8, Supplementary Material online; Root-Bernstein and Root-Bernstein 2019). Since in extant life, ribosomes are almost two-thirds rRNA, and only one-third proteins, a deeper understanding of rRNA evolution would help draw answers to the reductive coevolution of ribosomal proteins (Green and Noller 1997).

Since many signal transduction pathways are heavily dependent on transcription factors, it is essential to note that the COG system of annotation separates transcription factors and proteins involved in signal transduction. While some proteins have both [K] transcription and [T] signal transduction mechanism annotations, in our study, we have separated these annotations and counted them individually for an organism (e.g., a protein annotated as KT is counted once as K and once as T). Similar to the observations using COG annotations by Cordero and Hogeweg (2007), we did not observe quadratic scaling for the category [K] transcription, which has been previously reported by studies that used GO-PFAM annotations (Molina and van Nimwegen 2009; Charoensawan et al. 2010). We observed near-linear scaling in archaea and bacteria and lenient sublinear scaling in eukaryotes (table 1). Complex transcription regulation mechanisms might explain the sublinear scaling in eukaryotes, as complexity often gives rise to more proteins participating in more functions (Charoensawan et al. 2010). A more detailed analysis of the role of noncoding DNA and chromatin in transcription regulation and a more comprehensive sample size of eukaryotic species would help us better understand scaling trends of transcription regulation in eukaryotes. We observe superlinear scaling in signal transduction, as discussed below.

**Fig. 2.**
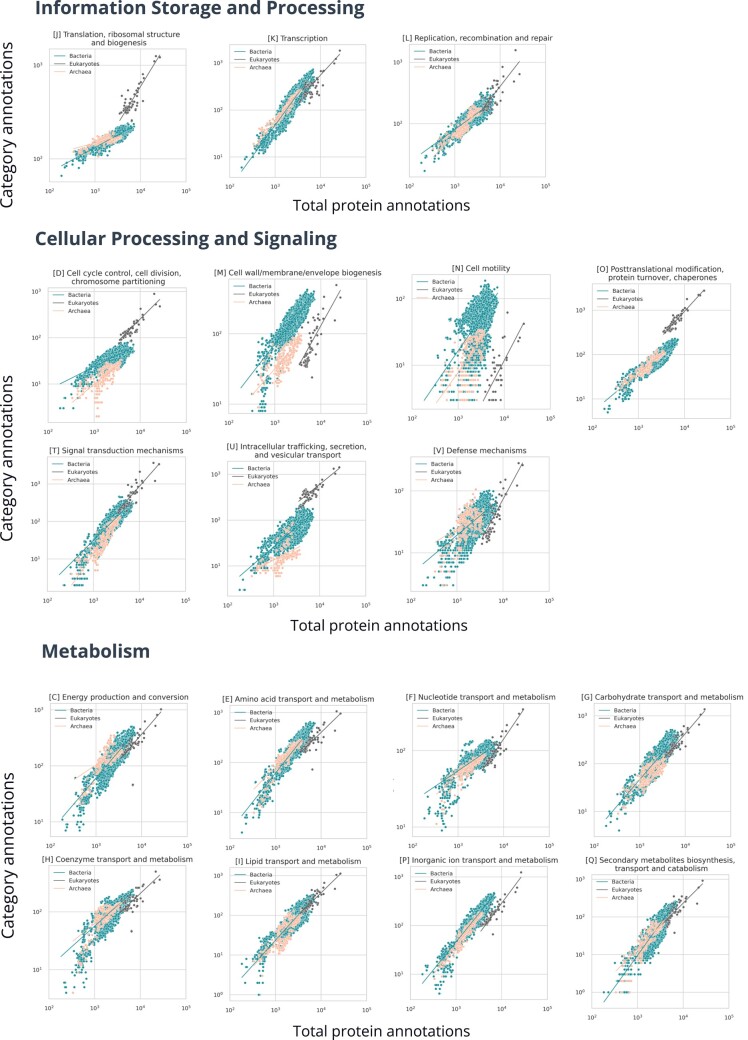
Overview of domain level scaling in protein classes. Axes are in log scale.

DNA replication allows organisms to pass on information, mutate, evolve, and adapt through evolution. Proteins in this category are required by the cell at specific time points in the lifecycle of a cell. They are well known to be similar between eukaryotes and archaea but different in bacteria (Koonin 2003). We highlight similarities and differences in their scaling patterns that have not previously been observed (Sclafani and Holzen 2007; Chia et al. 2010). Eukaryotes show almost linear scaling (0.92 ± 0.16) with genes involved in replication, recombination, and repair [L], while archaea show sublinear scaling, with the dilution being greater in smaller organisms (table 1 and fig. 2). Eukaryotes are believed to have not added unique subunits to the replisome machinery, and the subunits remain the same in the modern replisome machinery as the ancestral machinery (Chia et al. 2010). While we agree with this previous study considering eukaryotes, we differ in reporting that archaea scale sublinearly and do not show a continually increasing trend of unique subunits. Instead, the best fit is achieved by two lines of slope less than one, with smaller genomes scaling at 0.45 ± 0.08 and larger genomes being fit with an exponent of 0.87 ± 0.08. Possible reasons for differences from the Chia et al. (2010) study could be different annotation methods or the fact that the Chia et al. (2010) paper discusses three subunits of the DNA replication system: PCNA, RFCS, and MCM, while the COG category replication, recombination, and repair [L] includes more proteins and complexes. They also report the rate of the increase with respect to the total protein annotations in different terms as we use in this paper. Additionally, we observed that bacteria support a breakpoint at about one-fourth of the total protein annotations. The initial 25% of the data points show a sublinear scaling exponent of 0.72 ± 0.03, while the later data points scale even slower with an exponent of 0.48 ± 0.02 (table 1), opposite to the trend in archaea where the exponent increased.

Although archaea show scatter in [B] chromatin structure and dynamics, Asgard archaea do show a trend when looked at alone (1.24 ± 0.70), similar to what is seen in eukaryotes (0.83 ± 0.10), while bacteria show scatter and sublinear scaling with an exponent of 0.41 ± 0.04 (table 1; supplementary fig. S5.1 and table S1, Supplementary Material online). eggNOG includes annotations of proteins that carry out histone acetylation, acetoin utilization, certain chromatin remodeling proteins, and other kinase activity proteins in bacteria, although other nucleoid-associated proteins are not explicitly annotated (Travers and Muskhelishvili 2005), and more detailed analysis of bacterial chromatin organization is needed.

The spliceosome, considered among the most complex modern cell machinery, is believed to have evolved its complexity through neutral evolution (Gray et al. 2010; Lukeš et al. 2011). Due to this ratchet-like behavior, we might expect that scaling would be linear or superlinear; however, we observe a sublinear scaling trend for [A] RNA processing and modification in the 79 eukaryotes we considered (table 1). The much discussed constructive neutral evolution in RNA processing enzymes (Stoltzfus 1999, 2012; Muñoz-Gómez et al. 2021) would be a future target where genomes would be sampled from last eukaryotic common ancestor (LECA) across more complex eukaryotes to identify if scaling increases with total functional categories, or if complexity is related to phylogenetic position and evolutionary descent rather than number of total functional categories.

#### Cellular Processes and Signaling: COG Categories D, M, N, O, T, U, V, W, Y, and Z

Previously, van Nimwegen (2003) observed sublinear scaling for cell cycle proteins. In bacteria included in the current analysis, category [D] “cell cycle control, cell division, chromosome partitioning” data support a breakpoint from about near-linear scaling (0.82 ± 0.03) below 30% of the bacterial total functional categories and are sublinear (0.42 ± 0.03) after that (fig. 1 and table 1; supplementary table S3, Supplementary Material online). We observe near-linear scaling for archaea and eukaryotes, though archaea show an interesting cluster of genomes where total functional categories are uncorrelated with category [D] abundance (table 1 and fig. 2).

Membrane proteins in prokaryotes encompass about 20–30% of the proteins expressed, and an evolutionary leap from simple to complex cells may have meant increasing the number of membrane proteins owing to the increase in special membrane features and newer compartments in cells (Gould 2018). Proteins in category [M] “cell wall/membrane/envelope biogenesis” scale superlinearly for small bacterial genomes (2.82 ± 0.51) while showing a mild sublinear scaling after about only 10% of total protein annotations on the *x* axis (0.87 ± 0.02; supplementary table S3, Supplementary Material online). As total functional categories increase from the smallest end, more unique proteins are required, whereas less diversity is required toward the larger scale. The breakpoint analysis in archaea reveals the opposite trend, with the exponent increasing with the total number of annotations from 0.05 ± 0.13 with a high scatter to 1.17 ± 0.24. In eukaryotes, the scaling is superlinear (1.38 ± 0.23), and, interestingly, scaling after the breakpoint in archaea is similar to this value. It is tempting to speculate that this similarity in scaling could be related to eukaryogenesis and could represent a scaling perspective on how Asgard archaea could have consistent physiology with eukaryotes in connection with the proposal that they engulfed bacteria to form the eukaryotes we know today (Gould 2018).

In relation to a transition from Asgard to Eukarya, we observed a nearly linear scaling in [U] intracellular trafficking, secretion, and vesicular transport proteins for Asgard archaea (1.17 ± 0.53; supplementary table S1, Supplementary Material online). The endomembrane system that consists of the Golgi apparatus, endoplasmic reticulum, vacuoles, and vesicle traffic is conserved in all sizes of eukaryotes, indicating their conserved functioning from the LECA (Gould 2018), agreeing with our sublinear scaling exponents for category [U] in eukaryotes (0.71 ± 0.14), indicating a perhaps less expansive addition of newer proteins from LECA.

It is also interesting to observe that while category [M] supports a breakpoint for bacteria, it does not for category [U], hinting toward a possible universal scaling pattern for intracellular trafficking, secretion, and vesicular transport proteins across a range of bacterial cell sizes (supplementary table S3, Supplementary Material online). More exploration into specific scaling patterns of these two categories could unfold the mechanisms behind the evolutionary scaling of proteins involved in building the cell wall membrane/envelope biogenesis systems and the endosymbiotic theory for the origin of eukaryotes from mitochondria.

[T] signal transduction mechanisms scale superlinearly in archaea after a breakpoint, changing from 0.64 ± 0.30 to 2.08 ± 0.17. In eukaryotes, the scaling is also superlinear, whereas in bacteria, the slope is 1.72 ± 0.04 for bacteria below 40% of the total annotations on the *x* axis, and the trend takes a linear shift (1.11 ± 0.07) for the larger organisms in bacteria (Sela et al. 2019; supplementary table S3, Supplementary Material online). These observations differ from previous studies, which show a continuous trend in the scaling of signal transduction mechanisms and for bacteria hint at increased efficiency through evolution, with cells using the same unique subunits to create more complicated machinery for more complex organisms as evinced by the near-linear exponent after the breakpoint (Sela et al. 2019). For archaea, however, the case is very different, and category [T] is enriched during genome expansion supporting an alternative hypothesis as to how archaea are signaling.

We observed superlinear scaling in eukaryotes for proteins in category [V] defense mechanisms (1.61 ± 0.20; table 1). For bacteria, we observe an exponent of 1.17 ± 0.08 for organisms up to about 23% of the total protein annotations (supplementary table S3, Supplementary Material online). The scaling shifts to 0.62 ± 0.03 after that, and in archaea, the scaling is sublinear (0.78 ± 0.12). Defense genes in prokaryotes tend to cluster into “defense islands,” which are highly susceptible to gene shuffling and rapid evolution, and many of these islands’ genes are uncharacterized, hinting that a greater inspection of these clusters would give us a better overview of the evolution of prokaryotic defense strategies (Makarova et al. 2013). The higher exponent in eukaryotes is interesting to consider since the splicing machinery and defense mechanisms are often related (Koonin 2006; Irimia and Roy 2014). Tracing this back to LECA, it is believed that the group II retroelements from the proteobacterial cell infected the host archaeal genome (Koonin 2006; Irimia and Roy 2014). The host developed defense mechanisms against these introns; some were retained, some were lost, and some were newly developed (Koonin 2006). What Koonin describes as an “intron catastrophe” could have led to an increase in processes like ubiquitin signaling acting as defense mechanisms: previously offering different functions, these mechanisms were already present in early cells that explored these mechanisms temporarily to defend against external invading nucleic acids (Gray et al. 2010). Constructive neutral evolution events might also answer why random events like ubiquitination become fixed over evolution as one of the mechanisms under the big umbrella of defense mechanisms (Gray et al. 2010).

Environmental surfaces would have influenced the evolution of bacterial motility, and in contrast, cell enlargement must have been an influencing factor for eukaryotes (Miyata et al. 2020). We observe steep superlinear scaling in [N] cell motility for eukaryotes and Asgard archaea with scaling exponents above two, while bacteria and archaea show scaling exponents close to one highlighting a role for motility with large cell size (table 1; supplementary table S1, Supplementary Material online).

Through evolution, organisms must have increased their protein turnover rate to compensate for mistranslation and maintain proteome homeostasis (Kalapis et al. 2015). We report an almost linear scaling for proteins involved in [O] posttranslational modification, protein turnover, and chaperones for all domains.

#### Metabolism: COG Categories C, E, F, G, H, I, P, and Q

The relationship between metabolism and cell size has been extensively discussed in several works (Kleiber 1947; Kempes et al. 2012, 2016, 2017; Gagler et al. 2022). It has previously been observed that categories corresponding to metabolism scale almost isometrically with total functional categories, with metabolic networks tending to expand proportionally with total functional categories (Sela et al. 2019). We highlight the different scaling patterns observed here.

[C] energy production and conversion support a breakpoint for bacteria very early (before 10% of the total protein annotations), similar to other breakpoints in bacteria in our analysis (supplementary table S3, Supplementary Material online). The scaling is superlinear (1.34 ± 0.28), then slows to near-linear (0.98 ± 0.02). Archaea scale superlinearly until 42% of total protein annotations (1.52 ± 0.09; supplementary table S4, Supplementary Material online) and split into two groups after the breakpoint demonstrating intradomain variation in energy production scaling. This splitting into two groups is not captured in our current breakpoint analysis due to a methodological constraint and should be dealt with in future work. Eukaryotes scale with an exponent of 0.82 ± 0.18.

[E] amino acid transport and metabolism show superlinear scaling for smaller bacterial genomes (1.53 ± 0.14) and near-linear scaling for the larger ones (1.10 ± 0.02). Archaea show superlinear scaling for the first 28% of the total protein annotations, but the scaling falls to linear after that (supplementary table S4, Supplementary Material online), and for eukaryotes, the scaling is just below linear. [F] nucleotide transport and metabolism is sublinear across the domains, with a breakpoint detectable in bacteria. Perhaps this progressive dilution is related to very early nucleotide metabolism in the Last Universal Common Ancestor (LUCA), where after cellularization and genome growth, an expansion in nucleotide metabolism was not needed. [H] coenzyme transport and metabolism shows superlinear scaling for both smaller bacteria and archaea genomes and sublinear scaling for bacteria and archaea with larger genomes. Growth of a small genome seems to require enrichment of this category whereas eukaryotes with their larger genomes are sublinear. Category [I] lipid transport and metabolism is nearly linear for all three domains, providing an example of possible universality (supplementary table S3, Supplementary Material online).

[P] inorganic ion transport and metabolism shows similar scaling trends in all domains, existing as another example of possible universal scaling, and [Q] secondary metabolites biosynthesis, transport, and catabolism is superlinear for all domains but less steep in eukaryotes than in bacteria and archaea.

The unidentified category [S] function unknown scales superlinearly in bacteria indicating that the scaling exponents of other categories could shift in the future as these proteins are annotated and added to other categories. It also indicates that larger genomes in bacteria could have rarer proteins that do not have homologs and thus are not annotated. These issues are less present in eukaryotes where [S] scales sublinearly and large genomes have proportionally fewer unannotated proteins.

### Variability between Microbial Phyla

**Fig. 3.**
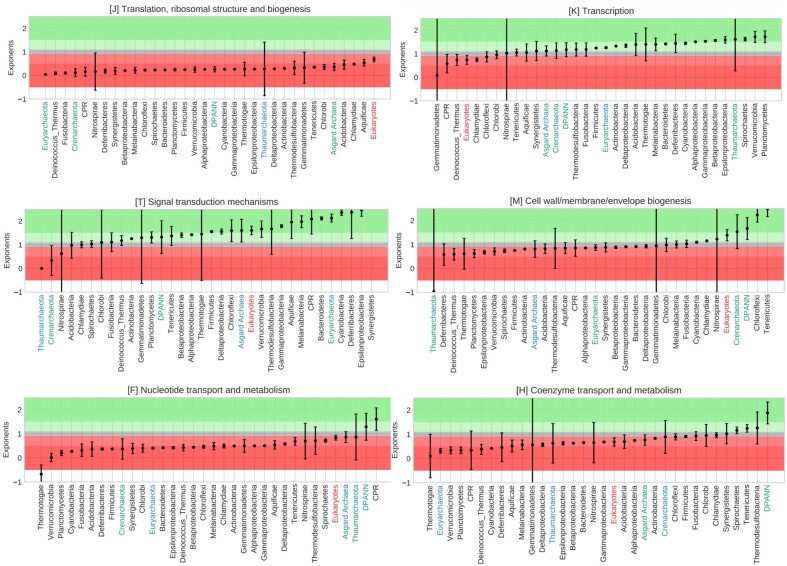
The spread of scaling between microbial phyla. Phyla arranged in the increasing order of their exponents with 95% confidence intervals for selected COG categories. Background horizontal span colors signify scaling, also evident from the scaling exponents on the *y* axis. From superlinear scaling (exponent more than one) to sublinear scaling (exponent less than one). The gray span in between (exponent close to one) signifies linear scaling. A full figure with all COG categories is available in the Supplementary material.

The observation that scaling relationships were best fit in many cases in bacteria and archaea by multiple lines led us to hypothesize that phyla-specific scaling relationships exist within the domains. We split up the domains into phyla to check how these individual phyla drive domain-level scaling patterns. Figure 3 shows the spread of scaling between microbial phyla for several COG categories (all of the COG categories are shown in supplementary fig. S4, Supplementary Material online). The figure also includes microbial Eukarya, which were not taxonomically separated due to fewer genomes.

Across the three domains, the ribosome is enriched in subunits from bacteria to archaea to eukaryotes with the highest number of subunits (Lecompte 2002). Category [J] translation, ribosomal structure, and biogenesis contains these proteins, and we find trends of phyla-specific reductive evolution and also some expansion. Eukaryotes show the closest exponent to one, and Asgard archaea (0.36 ± 0.15) fractionally dilute less compared with other archaea (0.14 ± 0.02; supplementary table S1, Supplementary Material online). In microbes, the number of ribosomes used increases with genome size due to proportionality between size and growth rate (Kempes et al. 2012, 2016), but since functional scaling is primarily sublinear, increased usage occurs by using more of the same thing and not an elaboration of types.

The scaling of regulation has received significant attention in past studies (Maslov et al. 2009; Molina and van Nimwegen 2009; De Lazzari et al. 2017; Sela et al. 2019). We observe phyla-specific scaling in categories [K] transcription and [T] signal transduction mechanisms, which are associated with regulation. Alpha-, Beta-, Gamma-, Delta-, and Epsilonproteobacteria show scaling with an exponent of around 1.5 (supplementary table S2, Supplementary Material online). Additionally, Planctomycetes, Spirochaetes, and Verrucomicrobia show high scaling exponents in addition to the members of Proteobacteria. All other phyla show exponents below 1.4 (supplementary table S2 and fig. S4, Supplementary Material online).

Deviating from the nearly universal trend of superlinear scaling in [T] are Chlamydiae and Spirochaetes, two groups notoriously known to be pathogenic, in addition to Acidobacteria (supplementary table S2 and fig. S4, Supplementary Material online). These three groups show linear scaling. Crenarchaeota and Thaumarchaeota in archaea show sublinear scaling (supplementary table S2 and fig. S4, Supplementary Material online). This is particularly interesting to note because Crenarchaeota use only one type of phosphorylation (Hanks type phosphorylation) and not two-component signal transduction like Euryarchaeota (Esser et al. 2016). Looking at these data, it appears that there is no universal scaling law of regulation.

DPANN and CPR show superlinear scaling for [F] nucleotide and [E] amino acid transport and metabolism (fig. 3; supplementary table S2 and fig. S4, Supplementary Material online). Figures 3 and 5 for [F] are of interest. As CPR and DPANN increase in unique protein annotations per total functional categories, this functional category might be among the first additions away from a minimal cell as they scale rather rapidly compared with the scaling of other proteins. DPANN shows an exceptionally high exponent for [H] coenzyme transport and metabolism, while CPR has an exceptionally high exponent for [I] lipid transport and metabolism (supplementary table S2, Supplementary Material online). Lipid biosynthesis pathways are not known for CPR (Castelle et al. 2018). Moreover, due to their exceptionally small sizes, CPR membranes could experience stress due to the high curvature-to-area ratio, explaining the abundant presence of lysolipids (Probst et al. 2020). CPR might also receive or forage certain lipids from surrounding bacteria in communities (Probst et al. 2020). However, the reason behind the high scaling exponent remains to be understood. These data make it intriguing to consider that there are multiple paths into, or out of, a reduced set of functional categories.

Tenericutes that lack a peptidoglycan cell wall, and Chloroflexi, which have a highly folded membrane but are somewhat enigmatic in terms of their membrane composition (Sutcliffe 2011; Skennerton et al. 2016), showed quadratic scaling of proteins involved in [M] cell wall, membrane, and envelope biogenesis (Sutcliffe 2010; Tocheva et al. 2016). Tenericutes also drive the drastic superlinear scaling in the initial part of the bacterial domain scaling plot (fig. 6). DPANN are superlinear in [M], again contrasting to CPR, which are sublinear.

[Q] secondary metabolites biosynthesis, transport, and catabolism show universal superlinear scaling trends except for three phyla, which also have a wide confidence interval (supplementary table S2, Supplementary Material online). Category [Q] contains a number of key catabolic proteins including the oxygenase cytochrome P450, numerous dioxygenases, SAM-dependent methyltransferases, acyl carrier protein, and microcompartment proteins. Conserved superlinear scaling across phyla for this group of proteins suggests that wider repertoires of catabolic proteins are required as genomes increase in size.

[N] cell motility is highly variable across microbial phyla. Although eukaryotes and Asgard are still among the highest scaling exponents, other groups stand out, including Cyanobacteria, Verrucomicrobia, Synergistetes, and Firmicutes (supplementary fig. S4, Supplementary Material online; table 2). Opposite to these members that have superlinear scaling are Thermatogae, Nitrospira, Melainabacteria, and Thaumarchaeota, which have exponents less than one. At least 18 motility systems exist (Miyata et al. 2020), although utilization of these systems as they scale with total functional categories is very diverse.

**Table 2:** Similarities and Differences between Functional Scaling within Archaea and Bacteria in Comparison to Asgard Archaea and Eukaryotes

Category	Archaea and Bacteria	Asgard Archaea and Eukaryotes
[L] replication, recombination, and repair	Sublinear	Linear
[N] cell motility	Near-linear	Quadratic (>2)
[O] posttranslational modification, protein turnover, chaperones	Sublinear	Linear
[F] nucleotide transport and metabolism	Sublinear	Sublinear
[H] coenzyme transport and metabolism	Sublinear	Sublinear
[Q] secondary metabolites biosynthesis, transport, and catabolism	Superlinear	Superlinear

[D] cell cycle control, cell division, chromosome partitioning shows near-linear to sublinear scaling across the tree of life (supplementary table S2 and fig. S4, Supplementary Material online). Planctomycetes are among the lowest scaling exponents and lack the multicomplex divisome most bacteria use for binary fission (Rivas-Marin et al. 2020). While the budding mechanism in bacteria of this phylum is not well understood, they use an extremely conserved component of the divisome (Rivas-Marin et al. 2020).

### Variability between Microbial Phyla: *Z*-Statistic Score

**Fig. 4.**
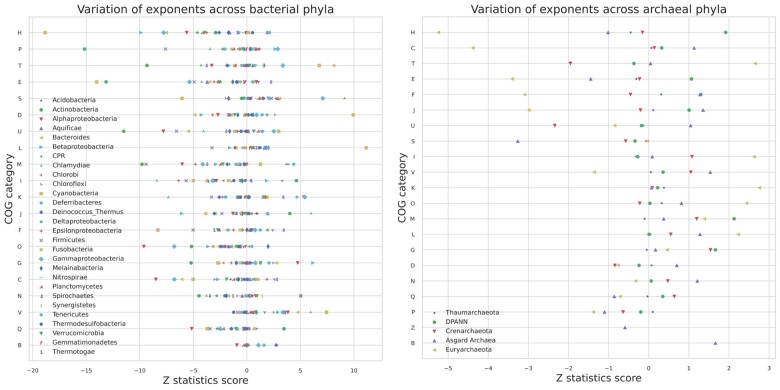
*Z*-Statistic plots showing the difference between scaling exponents of individual phyla from their respective domains.

Considering the expanded taxonomy in this study, the prominently varying scaling laws may highlight the differences in the physiology and architecture of prokaryotes. To quantify the deviation of the individual phyla exponents from the scaling exponents observed at the domain level, we calculated *Z*-statistic scores as before (Molina and van Nimwegen 2009). Figure 4 arranges the COG categories by the range of the *Z*-statistic scores in each category that were calculated for bacteria and archaea separately. These summarize how individual phyla/clade exponents differ from their respective domains (archaea and bacteria).

First, we can see that archaea and bacteria are unique in how variation is distributed across functional categories (fig. 4; supplementary table S6, Supplementary Material online). For bacteria, [H] coenzyme transport and metabolism showed the most variation (fig. 4; supplementary table S6, Supplementary Material online). The smallest variation was observed in [B] chromatin structure, [Q] secondary metabolites biosynthesis, transport and catabolism and dynamics, and [V] defense mechanisms, indicating that the bacterial domain exponents are a good representative of the microbial diversity for these categories. Furthermore, Actinobacteria are outliers. Cyanobacteria show extreme trends too. The *Z*-score of Gemmatimonadetes primarily lies near zero, indicating exponent values similar to the whole domain. Although these scores allow us to summarize the variability or similarity in exponents, it is important to note that the *Z*-score does not include the sample size of the groups, and there is no means of identifying the confidence intervals.

Among archaeal phyla, Euryarchaeota are significant outliers (supplementary table S6, Supplementary Material online). We hypothesize that this could be explained by a “slow scaling hypothesis,” which considers that as the genome size increases, there may be increased “protein moonlighting” (Copley 2012), or lower metabolic diversity in this group. Thaumarchaeota lies mainly near zero (supplementary table S6, Supplementary Material online). As with bacteria, [H] coenzyme transport and metabolism shows the most significant variation indicating the different physiological and functional organization of this category across phyla.

Lineages differ with respect to their functional repertoires (Watson et al. 2021), and we have demonstrated in this section how variable scaling is between phyla. These observations follow van Nimwegen et al. in showing that functional scaling is also unique to particular groups. Interestingly, when looking at figures 2 and 4, we observe that metabolism is the most similar between domains (data clouds overlapping on top of each other) but are some of the most variable categories between phyla (from *Z*-scores).

### Evolutionary Edges: Asgard Archaea, DPANN, and CPR

Organisms with small genomes like DPANN and CPR that lack major metabolic pathways show markedly high scaling exponents for proteins belonging to nucleotide and amino acid transport and metabolism (Castelle et al. 2018; fig. 3; supplementary fig. S4 and table S2, Supplementary Material online). This could be explained by the large variety of cellular activities that the proteins in these categories carry out in contrast to categories of the central dogma of biology (e.g., J) that have rather specific roles. It is interesting to note how CPR shows sublinear scaling in category [H] coenzyme transport and metabolism, while DPANN shows almost quadratic scaling in this category (fig. 3; supplementary table S2, Supplementary Material online), suggesting major differences between functional repertoire adjustment during genome expansion or shrinkage in these two very different phylogenetic groups. In addition to [H], CPR and DPANN show different scaling patterns in [C] energy production and conversion, [I] lipid transport and metabolism, [K] transcription, [M] cell wall/membrane/envelope biogenesis, [T] signal transduction mechanisms, and [U] intracellular trafficking, secretion, and vesicular transport while being similar in the rest of the categories. Although it is tempting to think of CPR and DPANN as phylogenetically “mirror images” of life at a small cell size with obligate cellular relationships, they are achieving their lifestyles in different ways.

**Fig. 5.**
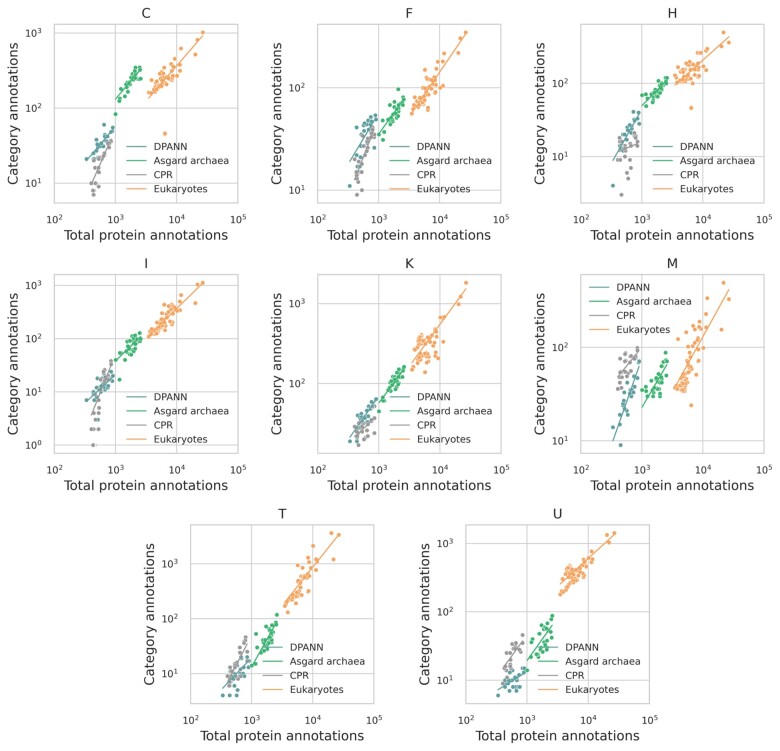
Scaling comparison showing the differences and similarities between the groups with small genome sizes (CPR and DPANN) and groups evolutionarily related (Asgard archaea and eukaryotes).

These patterns are shown for a subset of COG categories in figure 5 and for all the COG categories in supplementary figure S5, Supplementary Material online. Asgard archaea and eukaryotes have similar scaling exponents for all categories except [J] and [K] (table 2; supplementary table S2 and fig. S5, Supplementary Material online).

### Variability of Scaling within Microbial Phyla

**Fig. 6.**
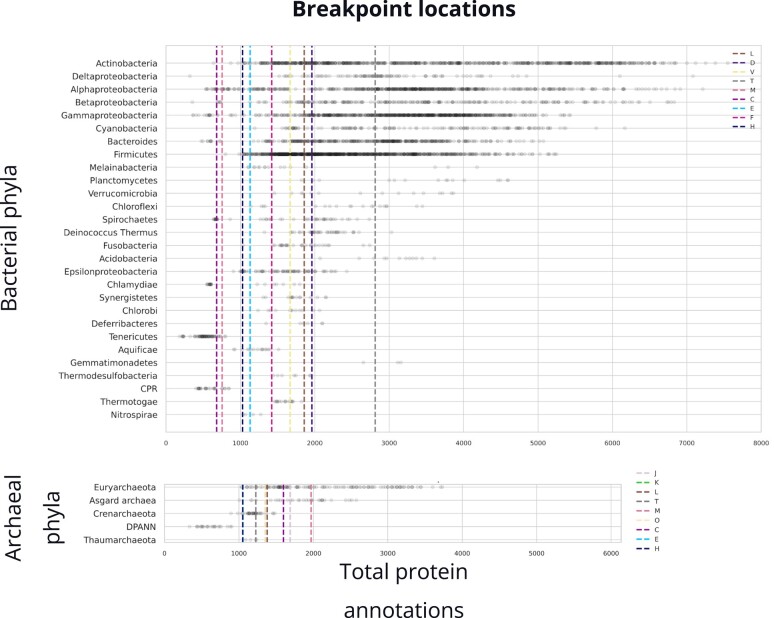
Breakpoint locations across bacterial and archaeal genomes showing that phylogenetic groups can traverse scaling exponents when their member genomes grow or shrink. Individual data points are total protein annotations of organisms. Phyla ordered from the most spanning to the least spanning on the *x* axis from top to bottom. For archaeal phyla, categories E and K are overlapped by categories H and O respectively.

We hypothesized that phyla-specific scaling might explain the observation of breakpoint analysis (fig. 1). However, when we overlaid the total protein annotations in each phylum with the breakpoint locations, we found that breakpoints are spanned by phyla. Since phyla are not restricted to one side of a breakpoint, it appears that phylogenetic groups can traverse scaling exponents when their member genomes grow or shrink (fig. 6).

Both archaeal and bacterial annotations show a breakpoint in categories [C] energy production and conversion, [E] amino acid transport and metabolism, [H] coenzyme transport and metabolism, [M] cell wall/membrane/envelope biogenesis, [T] signal transduction mechanisms, and [L] replication, recombination and repair.

The position of the breakpoints is observed to vary between archaea and bacteria domains, and the total protein annotations harbored by phyla genomes seem to be flexibly positioned on top of these breakpoints. For the metabolism category [C], while bacteria show a breakpoint toward the extreme low end, archaea have this breakpoint positioned near the center. For [E] and [H], archaea and bacteria both have breakpoints positioned around the same number of annotations. The trends in the scaling before and after the breakpoints, as discussed in detail in the Domain Level View of Functional Scaling section above, are similar in both bacteria and archaea, with the scaling slowing down from superlinear scaling after the breakpoint and the slowing down being more prominent in [C] and [H] for both domains.

For cellular processing and signaling categories [M] and [T], the breakpoint locations are different between archaea and bacteria with the breakpoint for [M] lying near the lower end for bacteria and the higher end for archaea. For [T], the breakpoint falls near the higher end for bacteria, while it is centered for archaea, crossing all phyla except for DPANN. The patterns in the scaling are very different too. While [M] and [T] both scale superlinearly before the breakpoint for bacteria, they scale sublinearly for archaea. After the breakpoint, these categories scale sublinearly for bacteria but superlinearly for [T] and nearly linearly for [M] archaea.

Most of the breakpoints occur near the smallest numbers of functional categories. This makes sense since a variety of dramatic physiological and compositional shifts are known to occur for the smallest cells (Kempes et al. 2012, 2016, 2017). For example, growth rates, ribosomal abundances, and the space available for macromolecules all have asymptotic behavior for the smallest cells (Kempes et al. 2012). The smallest cells are limited in internal space for macromolecules and metabolism shifts toward maintenance purposes, leaving little for growth. These dramatic changes impose serious constraints that are likely to be accompanied by corresponding shifts in the genome scaling as genomes are reduced.

Breakpoints for categories [D] cell cycle control, cell division, chromosome partitioning, [V] defense mechanisms, and [F] nucleotide transport and metabolism are unique to bacteria, and [J] translation, ribosomal structure and biogenesis, [K] transcription, and [O] posttranslational modification, protein turnover, chaperones are unique to archaea. Although we consider statistically significant differences in the position of the breakpoints, we must interpret cautiously and propose future work where understanding the evolutionary significance of breakpoints would need extensive analysis of specific phyla.

### Genome Scaling Is Not Correlated with the Phylogenetic Distance between Phyla

Phylogenetic distance between organisms is related to genome composition (Touchon et al. 2020; Prondzinsky et al. 2023) Given this, we hypothesized that variability in scaling might be related to phylogenetic distance between members across the tree of life. To investigate this, we used two trees of life constructed from conserved proteins (Hug et al. 2016; Martinez-Gutierrez and Aylward 2021). We calculated the patristic distance between clades to determine if scaling relationships among phyla are related to taxonomy evolution in both trees. No clear evolutionary relationship between phyla and groups was observed (supplementary fig. S3, Supplementary Material online), suggesting that variability in scaling is not related to the evolutionary relationships between taxonomic clades but instead may be related to environmental, physiological, or other variables. One interpretation for a lack of this correlation is that metabolism is quite modular, with the same proteins being used to connect or enable different metabolic modules or pathway flows. For example, Rubisco, the enzyme famous for carbon fixation in Cyanobacteria, is also found in Zetaproteobacteria, which couple CO_2_ fixation to iron oxidation and are phylogenetically very distant from each other.

### What Do Our Results Tell Us about the Earliest Genomes?

Understanding universality or variability in scaling patterns across diverse species would help us work our way backward to understand what to expect in early evolutionary periods, where cells similar to those today may have emerged from smaller genomes. Due to an inability to copy large amounts of information with high fidelity, it is thought that the first genomes may have been smaller than today's (Eigen and Schuster 1978; Woese 1998; Sharov 2006). On today's tree of life, the CPR and DPANN clades both show small genome sizes, which, together with branch position on some trees, have been used to suggest that these organisms might be ancient, though recent analyses suggest a revision of these branch positions and suggest evolution by derivation (Coleman et al. 2021; Martinez-Gutierrez and Aylward 2021; Moody et al. 2022). The genome composition of the LUCA is not clear (Berkemer and McGlynn 2020), and from this current analysis, it appears that functional expansion and reduction trends are not phylogenetic variables. Thus, it seems difficult to point to features of the earliest genomes with these data.

It is tempting to look at the scaling relationships presented here and consider where they might point to in terms of a minimal cell with a highly reduced genome. However, many of the scaling relationships are unique between the small CPR and DPANN, including the following: [H] coenzyme transport and metabolism, [C] energy production and conversion, [I] lipid transport and metabolism, [K] transcription, [M] cell wall/membrane/envelope biogenesis, [T] signal transduction mechanisms, and [U] intracellular trafficking, secretion, and vesicular transport (supplementary table S2 and fig. S5, Supplementary Material online). On the other hand, the other categories have the same scaling exponent between CPR and DPANN, suggesting that these might be universal to smaller cell sizes. Diversity in scaling between these groups tells us that there are multiple ways to scale from the small end of the total functional category range. Altogether, the scaling relationships of these groups could have implications for synthetic biology and guide an understanding of possible genome growth trajectories in very small cells.

Most of the previous scaling analyses of bacterial physiology focus on the connection between cellular rates, processes, abundances, and cell size (DeLong et al. 2010; Kempes et al. 2012, 2016, 2019). This is a natural connection, given that the scale of the system sets the values of various physical constraints. Such efforts have revealed how metabolic rates, growth rates, translational rates, and the abundances of all major macromolecules change with cell volume (Kempes et al. 2012, 2019). Genome size also changes with cell size systematically, which means that the relationships observed here also change with cell size, and this provides a way to compare how the abundance of functional categories changes with increasing size compared with how the overall physiology and macromolecular composition changes (Kempes et al. 2012, 2019).

Tracing back to the earliest cells, we are left with the question of how functional categories arrived in taxonomic clades: were they transferred horizontally from another group? or did they originate de novo within the group? In our analysis, functional groups associated with metabolic processes tend to have the highest variation in scaling exponents ([H] and [P] in fig. 4). Since COG categories associated with metabolism also have been transferred the most between the domains (Berkemer and McGlynn 2020), it may be that phyla-specific scaling patterns can be explained at least partially by the accretion of functions via horizontal gene transfer, though more detailed protein family–specific analyses are necessary to investigate repurposing/elaboration of existing genes versus alternative hypotheses of de novo birth and/or exaptation of other genes.

### Toward an Understanding of Evolution and Scaling in Biology

The relationships that we have described here in terms of the scaling of functions within genomes reveal fundamental regularities—and also variability—in genome organization. However, the underlying mechanisms behind these scaling relationships demand further explanation. Several simple mechanisms have been proposed for the scaling of certain categories. For example, the near quadratic scaling previously observed in regulatory genes suggested that every new gene addition to the genome comes with a pairwise consideration for regulation (van Nimwegen 2003; Ahnert et al. 2008; Molina and van Nimwegen 2008, 2009). Nevertheless, such proposed mechanisms have not been elucidated for every COG category, and as we have shown here, these scaling relationships can vary between phylogenetic groups. In fact, even the scaling of macrophysiology, such as growth rates, metabolic rates, and macromolecular concentrations, with cell or total functional category amount, does not give us a consistent way to explain the functional scaling relationships. For example, metabolic rate scales superlinearly with total functional categories in prokaryotes and sublinearly in eukaryotes (DeLong et al. 2010). This overall scaling and shift between prokaryotes and eukaryotes are mirrored by the metabolic categories [C], [E], [G], and [H], with higher scaling exponents in the former. However, ribosome and accompanying translational macromolecule intracellular abundances scale superlinearly with total functional categories, but COG category [J] scales very sublinearly where the exponent is closer to zero. This could be because the ribosomal machinery is so conserved that there is not much expansion in the diversity of similar genes within [J].

Variability in scaling between phyla has been previously attributed to a dependence of total functional categories on the ease of horizontal gene transfer or genome plasticity (Sela et al. 2019); our observation of variability of scaling factors between phyla may suggest that genome plasticity is broadly different between these groups. Although we can offer explanations related to the regulation scaling, we cannot yet explain the full variability of exponents. Genome size correlates with cell size, and cell size is a predictor of macrophysiology, so there is a hidden set of features that matter more than taxonomy.

While we include facets of scaling in biology not previously discussed—most notably our breakpoint analysis and lack of taxonomy relationship to scaling—several things deserve future attention, for example, copy number, for a more comprehensive understanding of evolution. Additionally, in our study, we focused exclusively on microbial organisms, and largely limited our analysis of phyla to the NCBI taxonomy; future work that includes multicellular organisms and the GTDB taxonomy will be valuable in parsing taxonomic patterns and linking functional scaling with cell and organism plan. The differences in scaling observed using different projections of gene function also need to be explained. Previous work has shown that EC categorization reveals universal scaling across the tree of life (Gagler et al. 2022) compared with the many shifts in scaling observed here for COG categories. This points to deep questions about evolution observed at the level of broad enzyme functionality (Gagler et al. 2022) that is not observed at the level of physiological functionality and homologous groups of proteins.

Variability in the fraction of nonannotated proteins in a given genome, the challenge of identifying open reading frames, and the difficulty in obtaining accurate groups of functionally equivalent homologous sequences all pose challenges for the type of study conducted here. Additionally, some taxonomic groups are undersampled making it difficult to analyze their contributions in driving the slopes of their respective domains. We expect this study to positively encourage further studies on scaling in biology as genome availability increases and annotations become more complete and thorough. We expect our understanding of functional scaling to deepen to help us ask more relevant questions about organism design and functional evolution.

## Materials and Methods

### Data Processing and Power Law Fitting

We considered COG annotations of sequenced genomes using the eggNOG database (Huerta-Cepas et al. 2019). We used the file sets bacteria and archaea on the eggNOG database downloads page http://eggnog5.embl.de/#/app/downloads. Using the eggNOG-mapper v2, we annotated the proteins of groups not available in the eggNOG database, like Melainabacteria, CPR, DPANN, Asgard archaea, and some unicellular eukaryotes using their protein FASTA files from GenBank Assembly to the eggNOG mapper. Asgard archaeal genome information was obtained from the Genome Taxonomy Database (GTDB; Parks et al. 2021; Rinke et al. 2021). We mapped the files to get their COG annotations from the eggNOG annotations. (eggNOG has protein annotations in the form of eggNOG IDs and the corresponding organisms with that particular eggNOG ID annotations. The eggNOG IDs correspond to specific COG annotations. All these are different files in the database, and the annotations in eggNOG have multiple COG categories defining a single eggNOG ID.) Further, we found the total number of annotations in a COG category and the total number of functional annotations in an organism. In cases where a protein had more than one COG annotation, these were split and counted as individual counts in the total number of functional annotations.

We used ordinary least square (OLS) regressions of the log-transformed data. We chose this method for easy intercomparison with various previous studies that employ this method. The 95% confidence interval on slope and intercept was found using *t* continuous random variable of scipy.stats.linregress from SciPy (Virtanen et al. 2020).

We carried out a null test by shuffling the COG category annotations. This resulted in orthogroups with very different abundances per organism, but the total number of COGs with categories remained the same. The power law fits for the shuffled data are in the Supplementary material (supplementary fig. S1, Supplementary Material online).

### Breakpoints

A challenge in scaling data is that often there is asymptotic behavior for the largest and smallest sizes in the system, which in some cases is anticipated from theory (Kempes et al. 2012, 2016, 2019). We used the piecewise_regression library (Pilgrim 2021) to compute the breakpoints. Within a taxonomic group, we present scaling analyses where either a single scaling relationship or two scaling relationships with a breakpoint are the best fits. We select the breakpoint model when it produces a significant improvement in the goodness of fit that we take to be a 5% decrease in the residual sum of squares (RSS) values compared with the single scaling relationship fit. It was calculated by dividing the RSS for one breakpoint by the RSS for no breakpoints (supplementary tables S7 and S8, Supplementary Material online). After finding the breakpoint locations, we fit the points using the same procedure as fitting the individual data plots. To visualize the locations of the breakpoints across genomes, we overlaid the breakpoint location against the genome spans of phyla (fig. 6; supplementary tables S3 and S4, Supplementary Material online).

#### Binning

OLS fits can be dominated by ranges of *x* values with the most data, which can lead to inaccurate power law fits. An uneven amount of data across the scales is definitely characteristic of our data. To deal with this unequal sampling, we binned the data set using scipy.stats.binned_statistics from SciPy (Virtanen et al. 2020) and then performed the OLS fit to the binned data. Supplementary figure S2, Supplementary Material online shows the results of the binned data and fits overlaid on the full data set, and supplementary table S5, Supplementary Material online compares the fit values where we only find negligible differences in some cases.

### *Z*-Statistics

We calculated the *Z*-statistic score to quantify the significance of the deviations of individual phyla exponents from their overall domain exponents using the formula developed by Molina and van Nimwegen (2009).


$$Z\mathrm{Score}=\frac{(\mathrm{Phylaslope}-\mathrm{Domainslope})}{\sqrt{{\mathrm{Phylaerror}}^{2}-{\mathrm{Domainerror}}^{2}}}.$$

For plotting, we consider both positive and negative deviations (fig. 4; supplementary table S6, Supplementary Material online). Error in the formula denotes the error bar for the slopes, found using *t* continuous random variable of scipy.stats.linegress from SciPy (Virtanen et al. 2020).

### Phylogenetic Distance Plots

We calculated the phylogenetic distances between phyla using the phylogenetic_distance_matrix() function from the Dendropy library (Sukumaran and Holder 2010) and the Hug et al. (2016) and Martinez-Gutierrez and Aylward (2021) tree of life. Since we did a phyla-level analysis, we took the average values of all the phylogenetic distances between all organisms in a phylum. To analyze the relationship between scaling exponents and phylogenetic distances, we used the absolute values of the differences between the scaling exponents of the respective phyla as well as the exponent quotients.

## Supplementary Material

evad214_Supplementary_Data

## Data Availability

Jupyter notebooks are available at https://github.com/riddhi7/functional-scaling-across-genomes
