# Supplementary material for: Scaling of Protein Function across the Tree of Life
Source: Genome Biol Evol. 2023 Nov 26;15(12):evad214. doi: 10.1093/gbe/evad214 (PMC10715193; doi:10.1093/gbe/evad214)
Supplement: evad214_Supplementary_Data [file evad214_supplementary_data.zip › GBE_SI_Figures.pdf]

Scaling of Protein Function Across the Tree of Life

Riddhi Gondhalekar[1,2,]*, Christopher P. Kempes[3]*, and Shawn Erin McGlynn[1,2,4,5]*

[1] Earth-Life Science Institute, Tokyo Institute of Technology, Ookayama, Meguro-ku, 152-8550 Tokyo, Japan

[2] School of Life Sciences and Technology, Tokyo Institute of Technology, Tokyo, Ookayama, Meguro-ku, 152-8550 Tokyo, Japan

[3] The Santa Fe Institute, Santa Fe, NM, USA

[4] Blue Marble Space Institute of Science, Seattle, WA 98154, USA

[5] Center for Sustainable Resource Science, RIKEN, 2-1 Hirosawa, Wako, 351-0198 Saitama, Japan

*** To whom correspondence should be addressed. Tel: +81-3-5734-2189; Fax: +81-3-5734-3416; Email: riddhig@elsi.jp**

Correspondence may also be addressed to CPK (ckempes@santafe.edu) and SEM (mcglynn@elsi.jp)

This document contains supplementary figures:

Supplementary Figure 1: Scaling in random orthogroups. Shuffled orthologs for a null test of the scaling exponents.
Supplementary Figure 2: Unibinned and binned power law fits.
Supplementary Figure 3: Phylogenetic distance plots.
Supplementary Figure 4: Phyla arranged in the increasing order of their exponents with 95% confidence intervals.
Supplementary Figure 5: Scaling comparison between CPR, DPANN, Asgard archaea, and Eukaryotes.

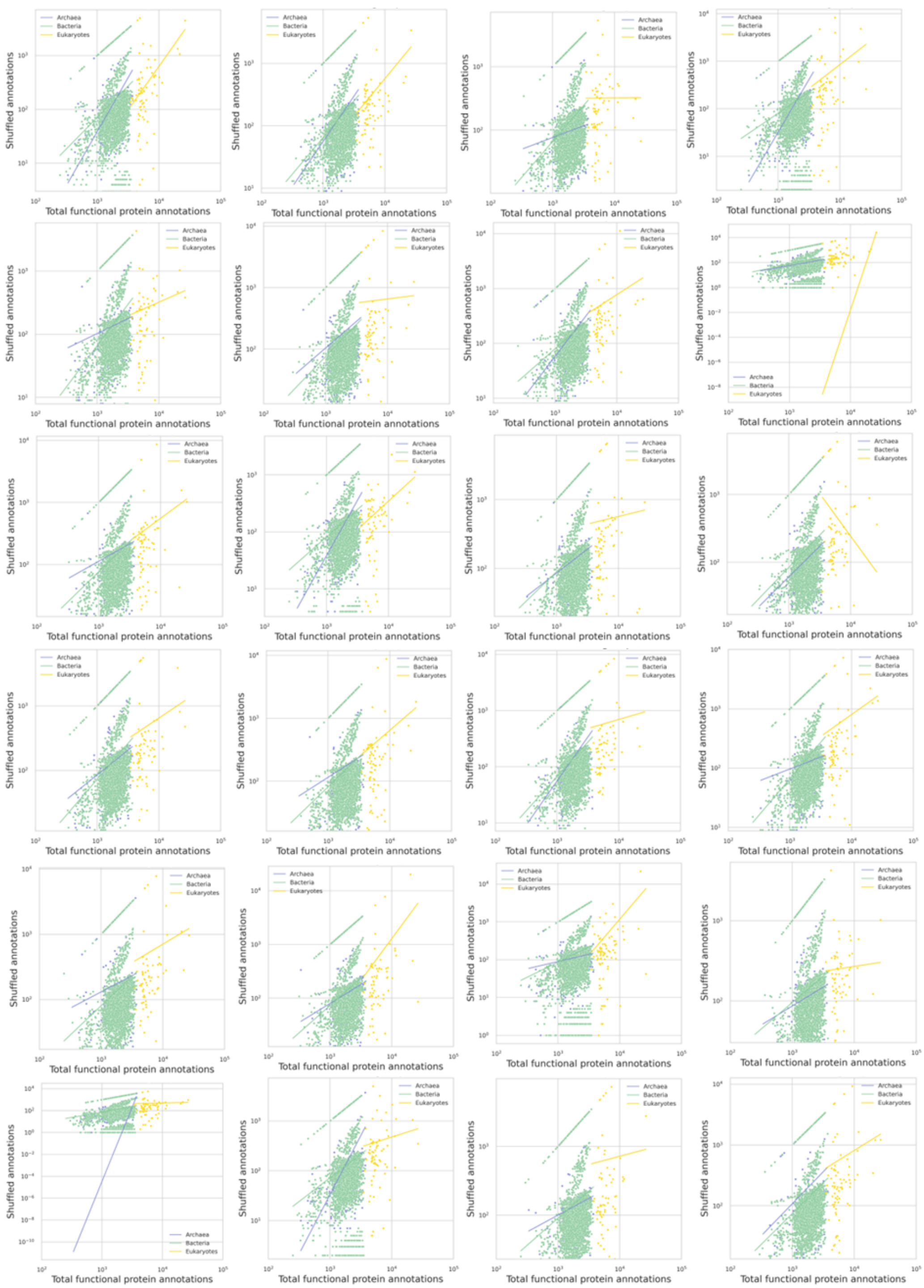

Supplementary Figure 1: Scaling in random orthogroups. Shuffled orthologs for a null test of the scaling exponents.

No specific patterns were observed when the orthogroups were shuffled.

1] Information Storage and Processing: COG categories A, B, J, K, and L

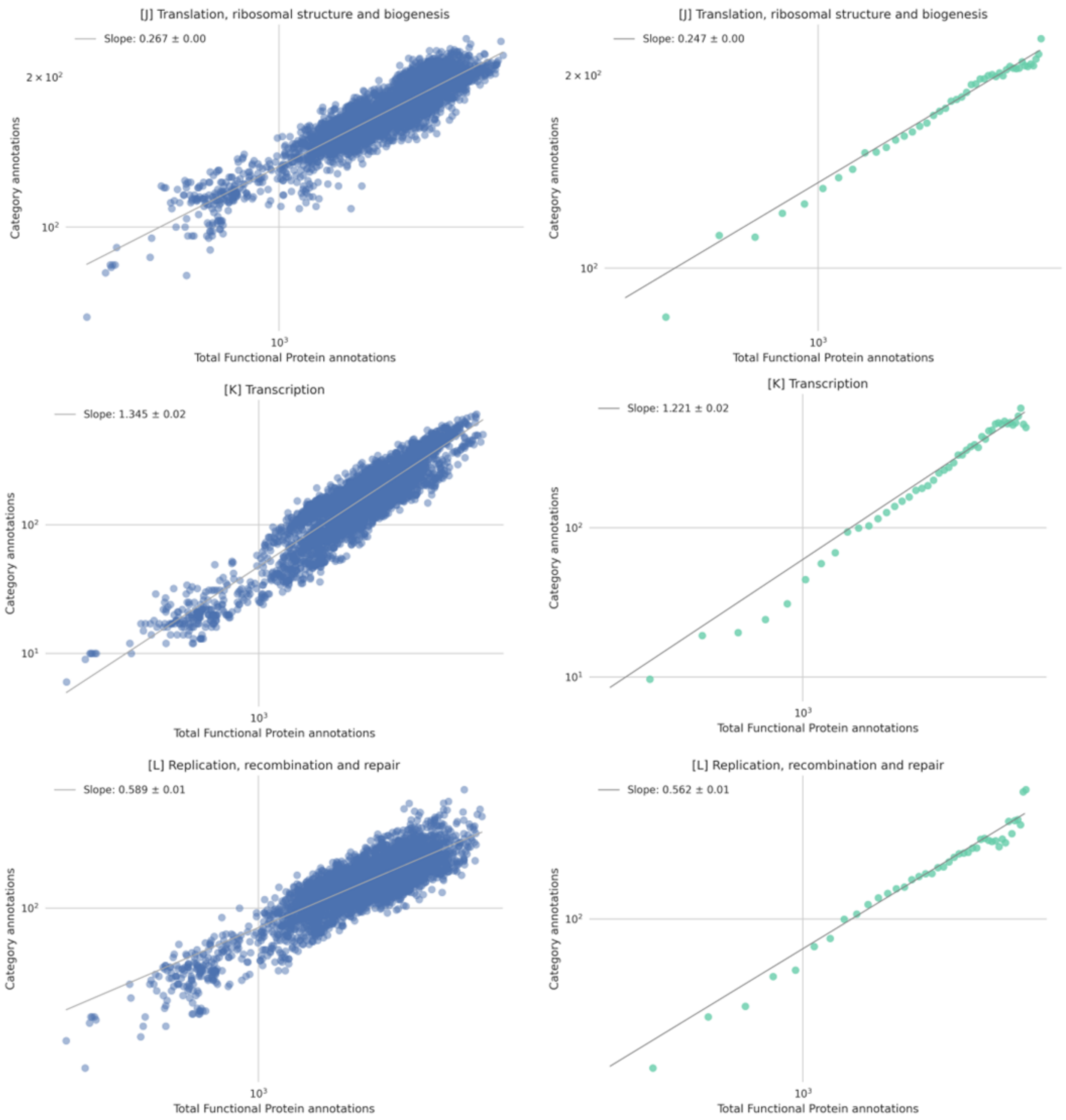

2] Cellular Processes and Signaling: COG categories D, M, N, O, T, U, V, W, Y, and Z

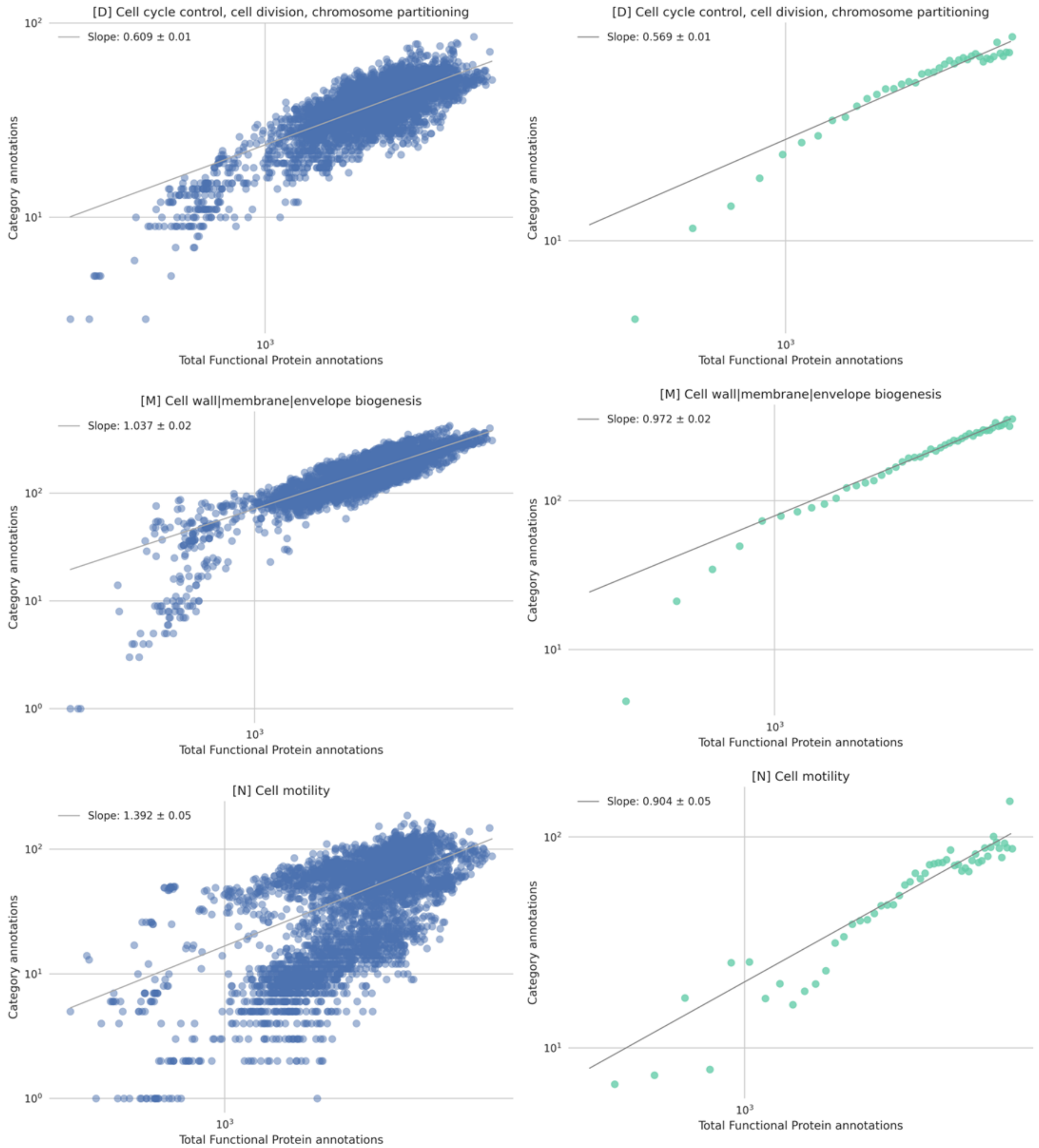

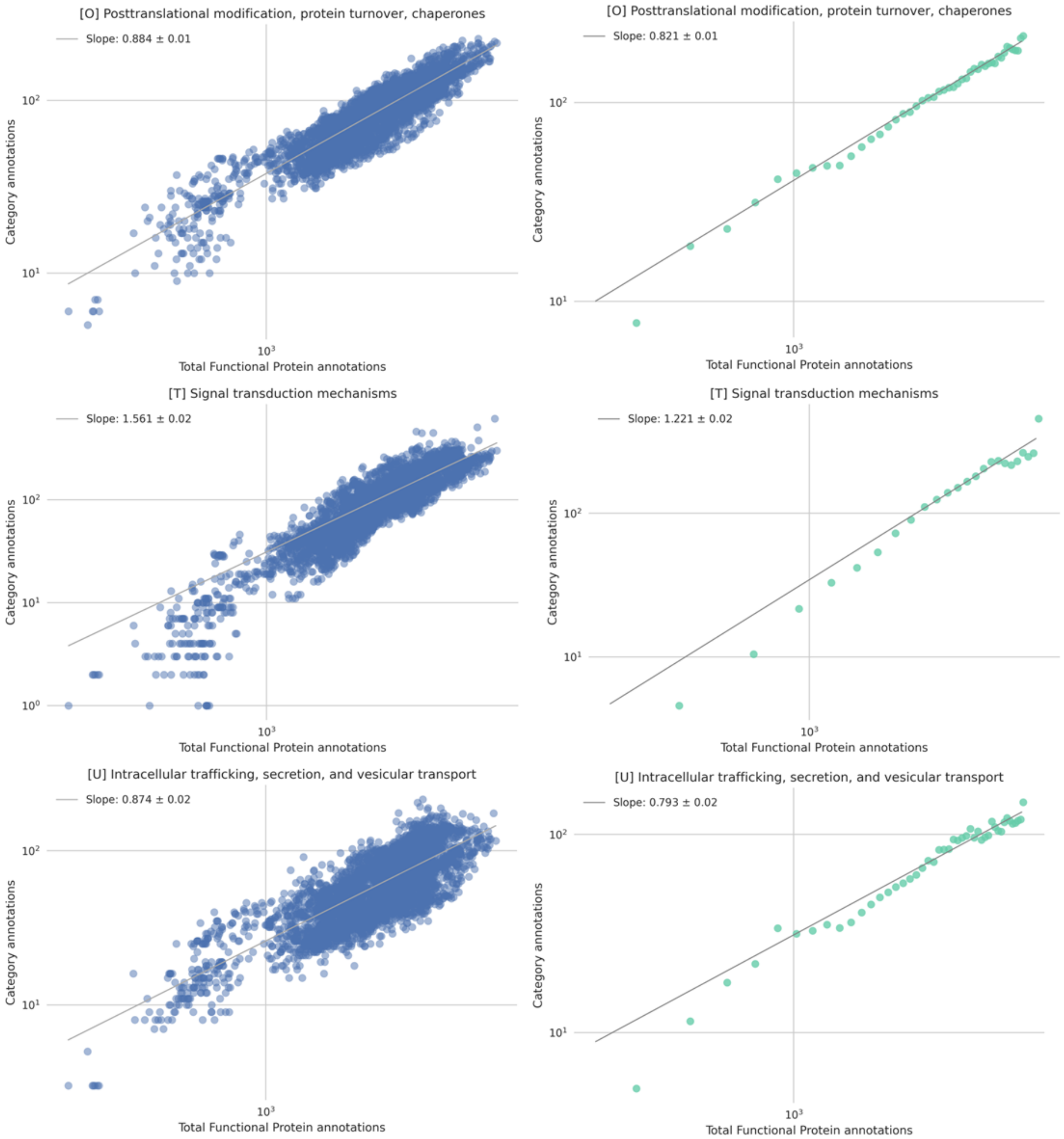

[O] Posttranslational modification, protein turnover, chaperones
Slope: 0.884 ± 0.01
Category annotations
Total Functional Protein annotations
[O] Posttranslational modification, protein turnover, chaperones
Slope: 0.821 ± 0.01
Category annotations
Total Functional Protein annotations
[T] Signal transduction mechanisms
Slope: 1.561 ± 0.02
Category annotations
Total Functional Protein annotations
[T] Signal transduction mechanisms
Slope: 1.221 ± 0.02
Category annotations
Total Functional Protein annotations
[U] Intracellular trafficking, secretion, and vesicular transport
Slope: 0.874 ± 0.02
Category annotations
Total Functional Protein annotations
[U] Intracellular trafficking, secretion, and vesicular transport
Slope: 0.793 ± 0.02
Category annotations
Total Functional Protein annotations

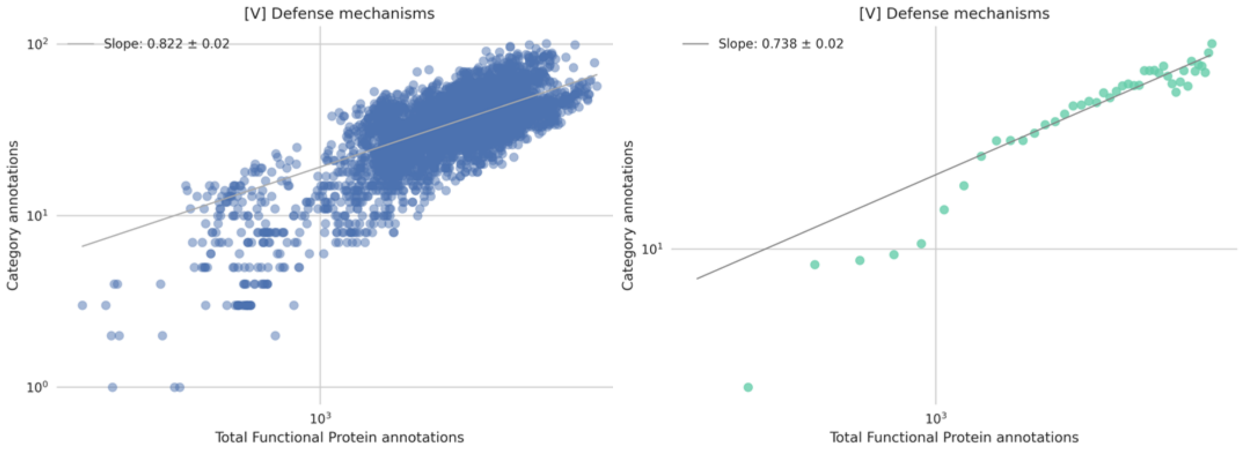

[V] Defense mechanisms
Slope: 0.822 ± 0.02
Category annotations
Total Functional Protein annotations
10^0
10^1
10^2
10^3
[V] Defense mechanisms
Slope: 0.738 ± 0.02
Category annotations
Total Functional Protein annotations
10^1
10^3

3] Metabolism: COG Categories C, E, F, G, H, I, P, and Q

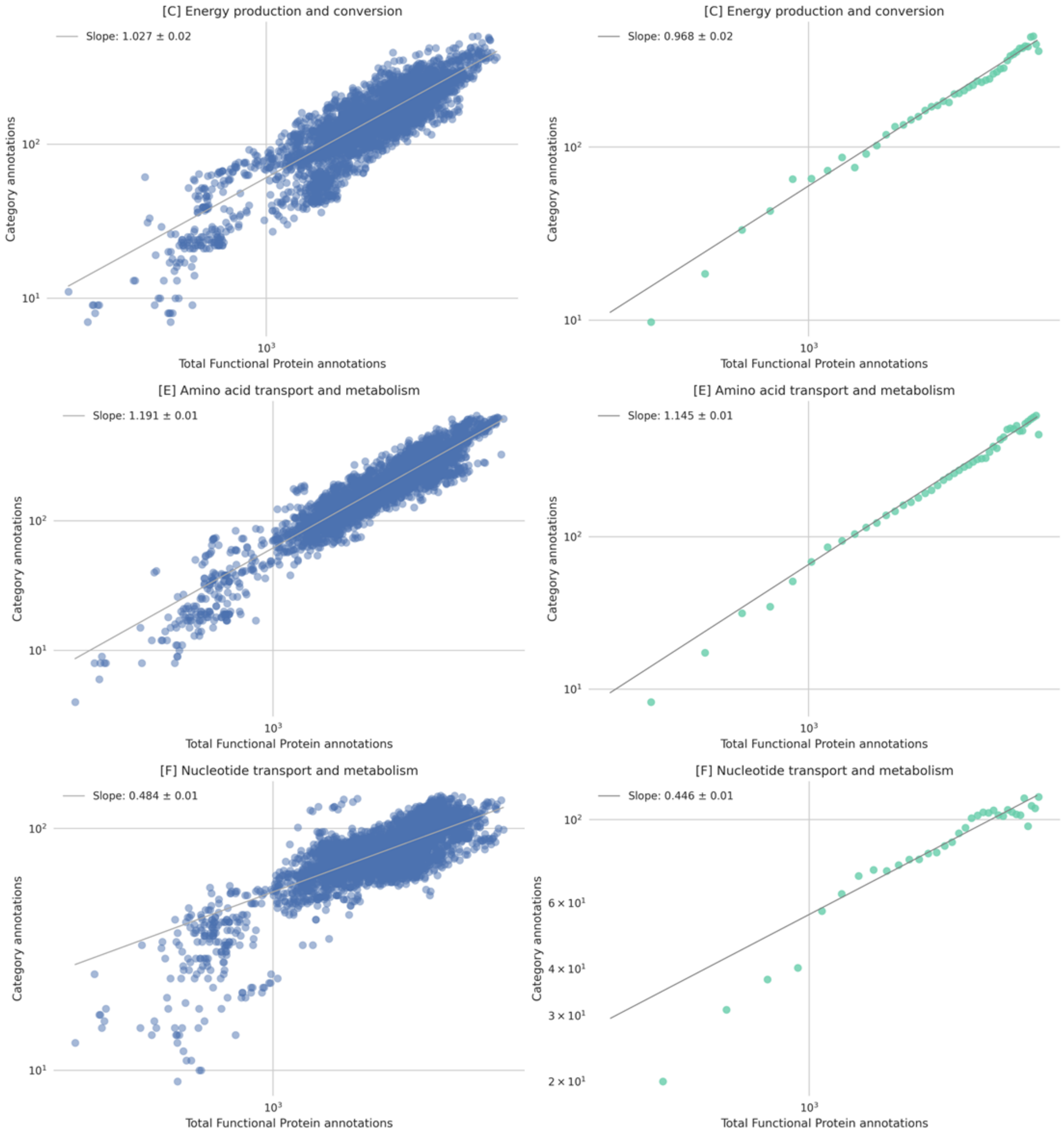

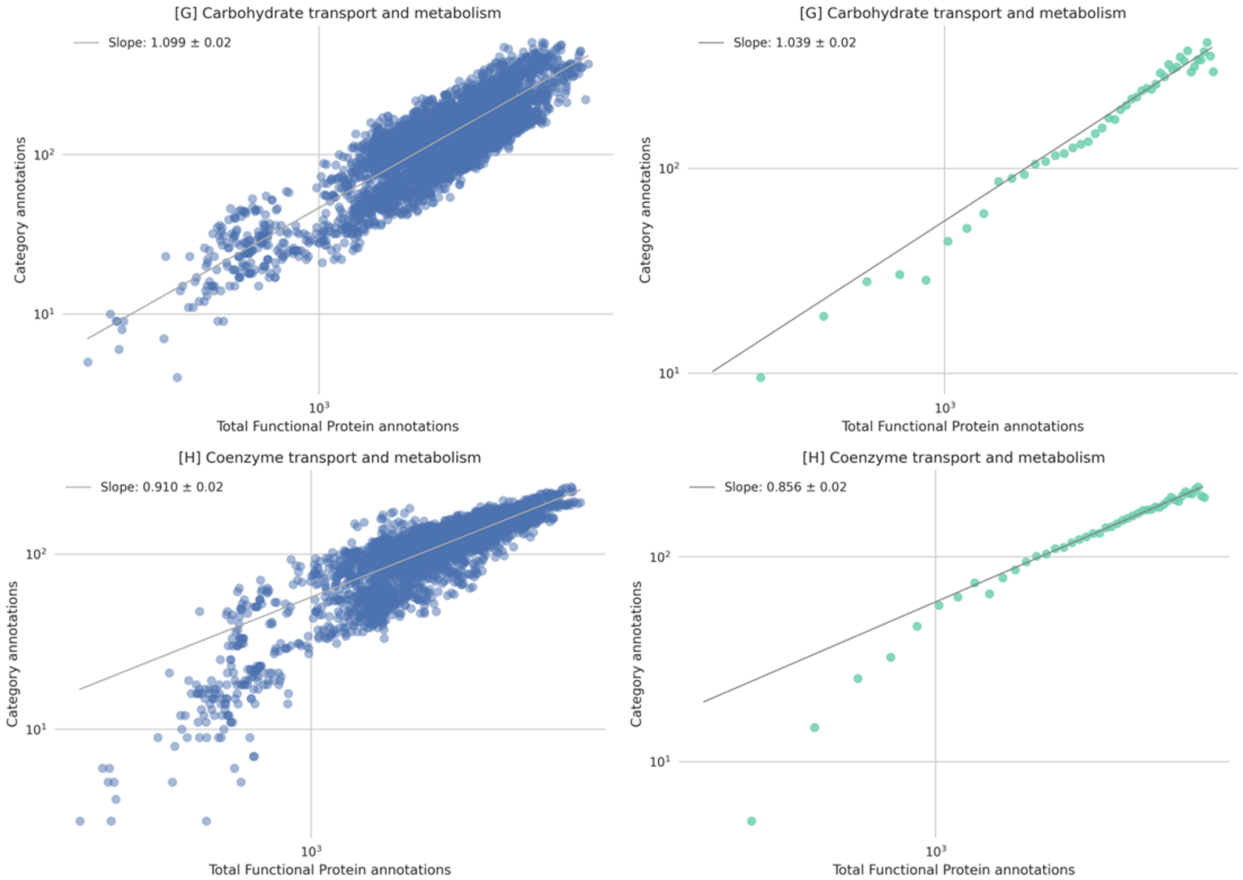
[G] Carbohydrate transport and metabolism
Slope: 1.099 ± 0.02
Category annotations
Total Functional Protein annotations
[G] Carbohydrate transport and metabolism
Slope: 1.039 ± 0.02
Category annotations
Total Functional Protein annotations
[H] Coenzyme transport and metabolism
Slope: 0.910 ± 0.02
Category annotations
Total Functional Protein annotations
[H] Coenzyme transport and metabolism
Slope: 0.856 ± 0.02
Category annotations
Total Functional Protein annotations

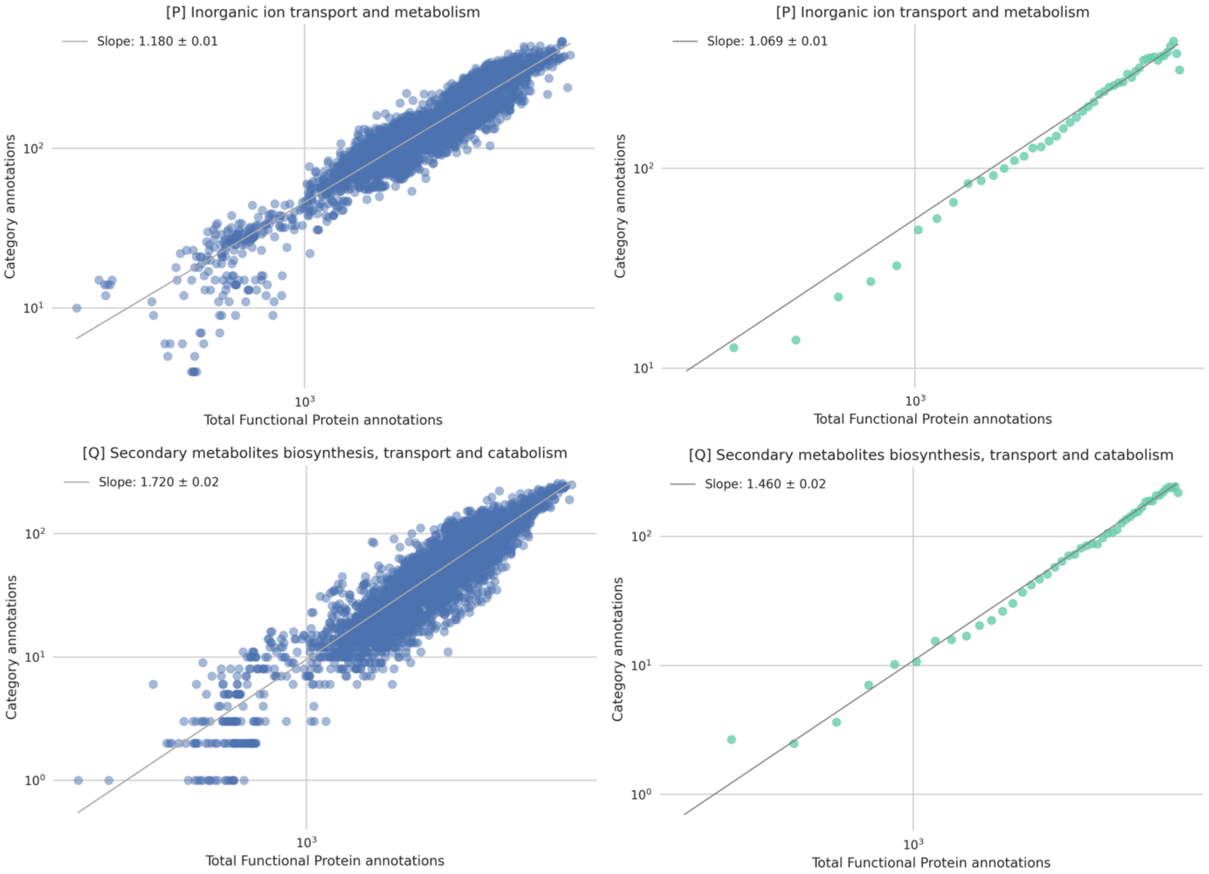

[P] Inorganic ion transport and metabolism
Slope: 1.180 ± 0.01
Category annotations
Total Functional Protein annotations
[P] Inorganic ion transport and metabolism
Slope: 1.069 ± 0.01
Category annotations
Total Functional Protein annotations
[Q] Secondary metabolites biosynthesis, transport and catabolism
Slope: 1.720 ± 0.02
Category annotations
Total Functional Protein annotations
[Q] Secondary metabolites biosynthesis, transport and catabolism
Slope: 1.460 ± 0.02
Category annotations
Total Functional Protein annotations

[S] Function unknown

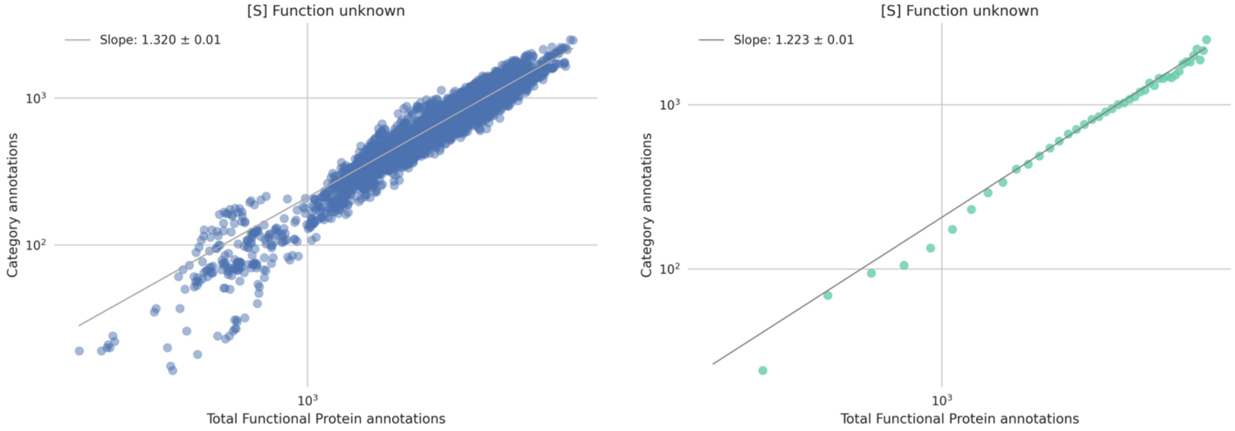

Supplementary Figure 2: **Unbinned and binned power law fits.** Axes are in log scale.

I. Phylogenetic tree from Hug et al. 2016

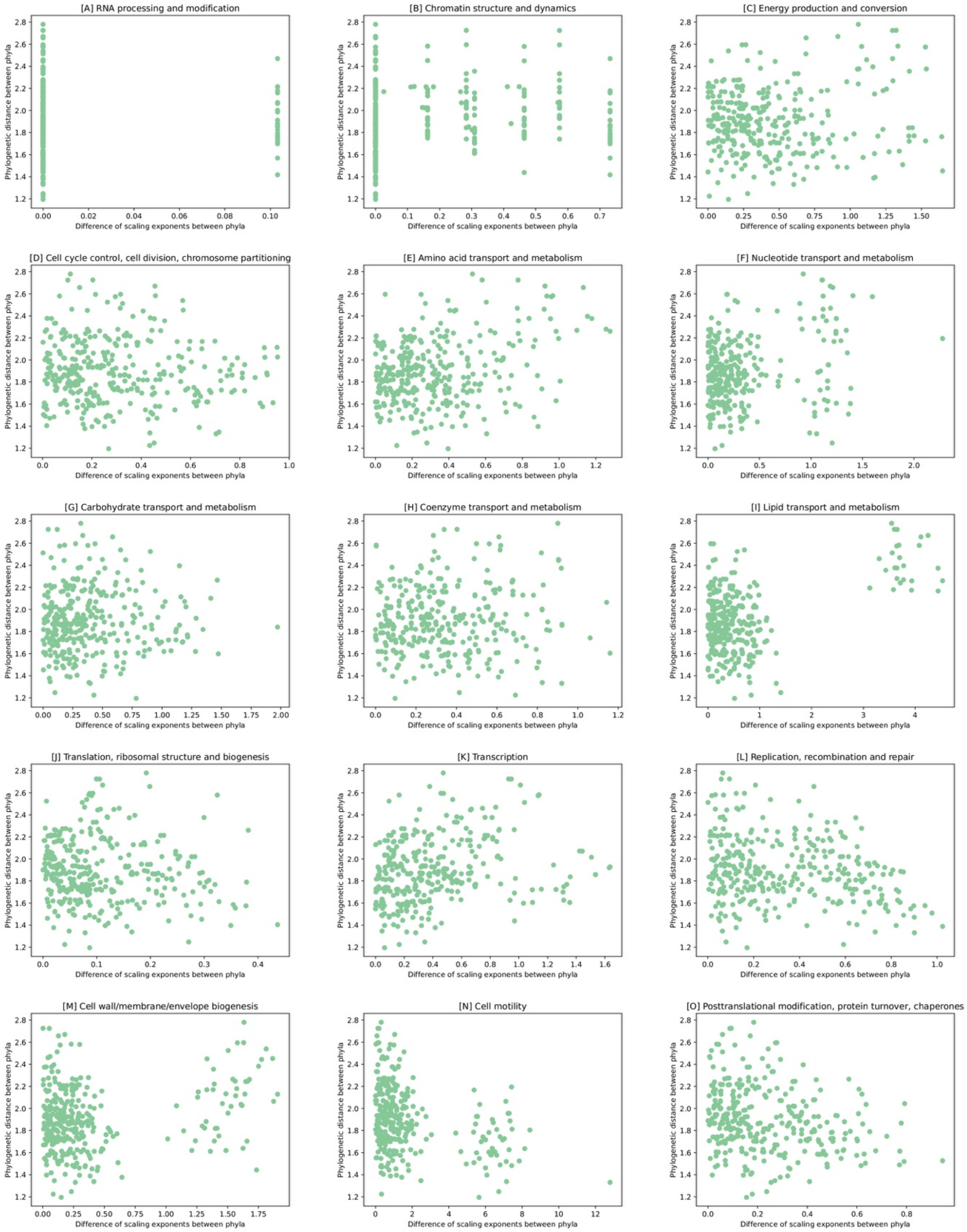

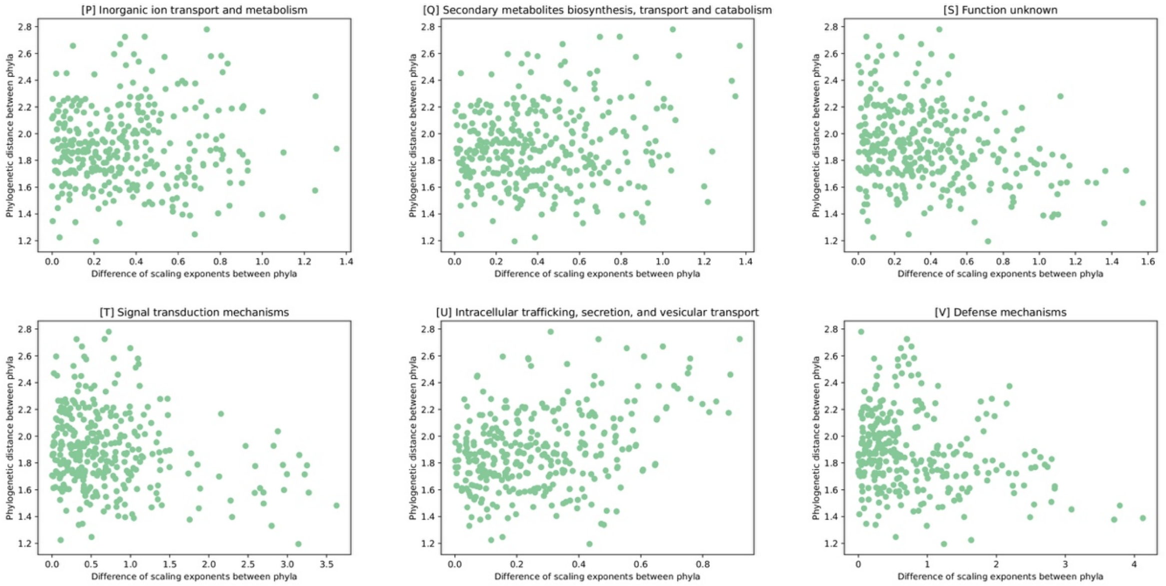

II. Phylogenetic tree from Martinez-Gutierrez and Aylward 2021

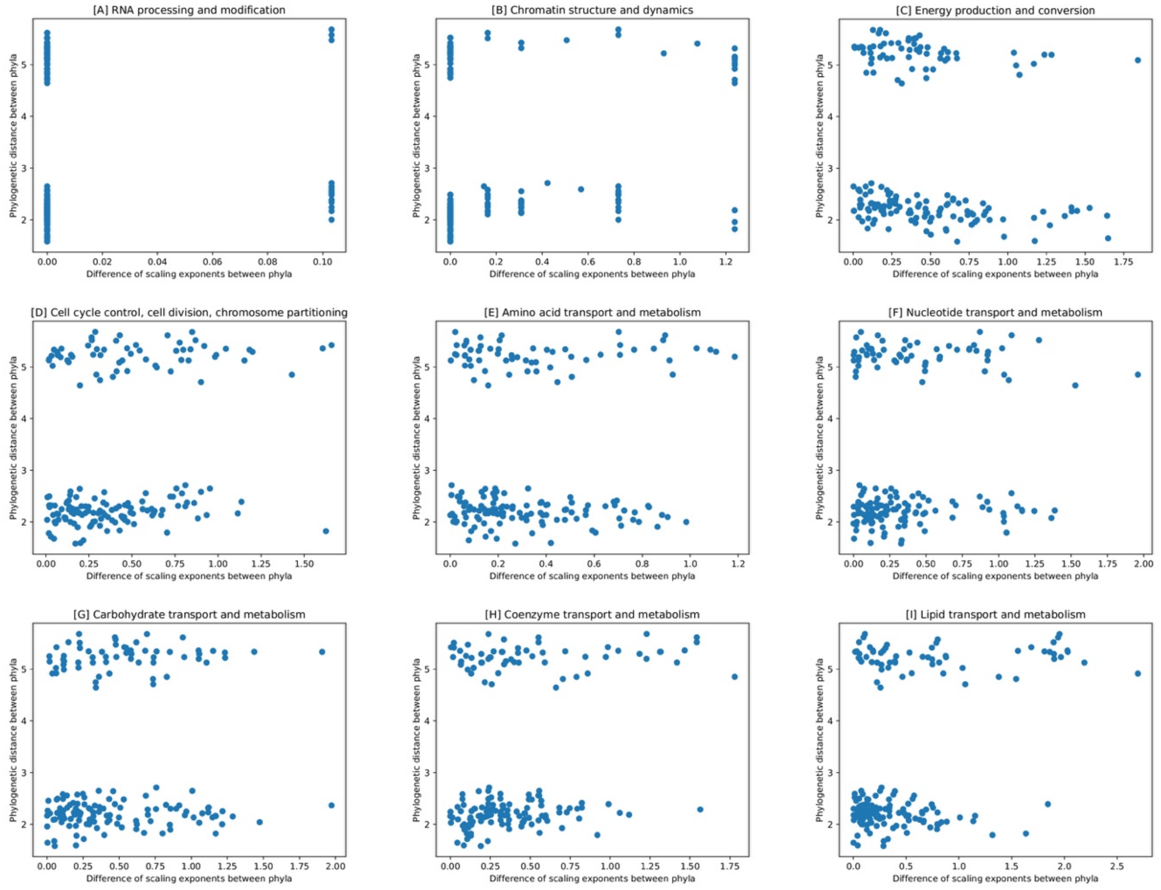

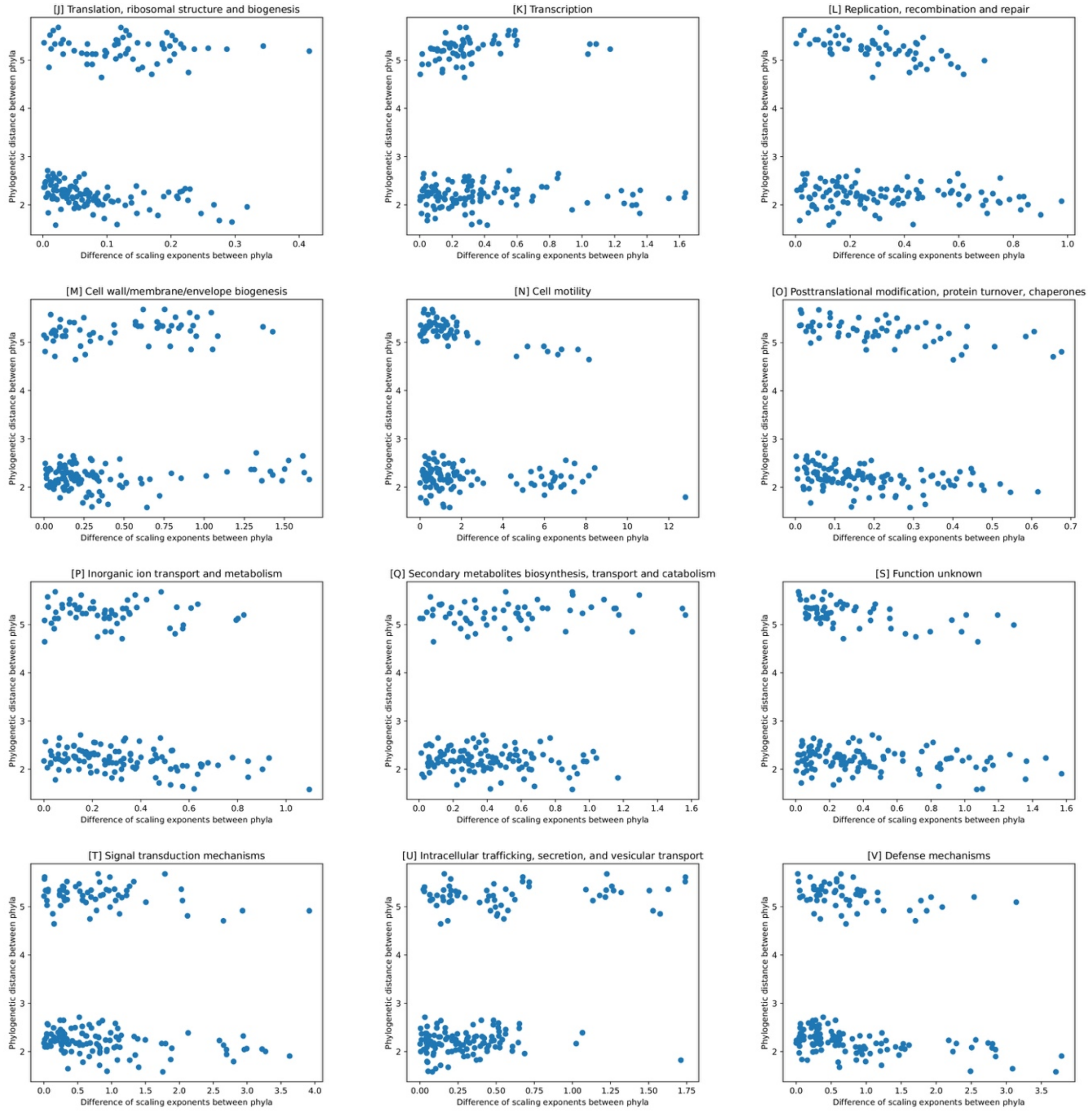

Supplementary Figure 3: Phylogenetic distance plots. X-axis is the absolute value of the difference between the exponents of the respective phyla.

The differences in taxonomy nomenclature between the NCBI database and the GTDB database were handled as given in the following table.

NCBI Taxonomy	GTDB Taxonomy
Euryarchaeota	Halobacteria
Euryarchaeota	Methanobacteriota
Euryarchaeota	Thermoplasmatota

Crenarchaeota	Thermoproteota
DPANN	Iainarchaeota
DPANN	Nanoarchaeota
Asgard archaea	Asgardarchaeota
CPR	Patescibacteria
CPR	KSB1
Deltaproteobacteria	Myxococcota
Deltaproteobacteria	UBA10199
Epsilonproteobacteria	Campylobacterota
Chloroflexi	Chloroflexota
Firmicutes	Firmicutes
Actinobacteria	Actinobacteriota
Acidobacteria	Acidobacteriota
Nitrospirae	Nitrospirota
Deferribacteres	Deferribacterota
Bacteroides	Bacteroidota
Gemmatimonadetes	Gemmatimonadota
Planctomycetes	Planctomycetota
Verrucomicrobia	Verrucomicrobiota
Spirochaetes	Spirochaetota
Cyanobacteria	Cyanobacteria
Thermotogae	Thermotogota
Synergistetes	Synergistota

1] Information Storage and Processing: COG categories A, B, J, K, and L

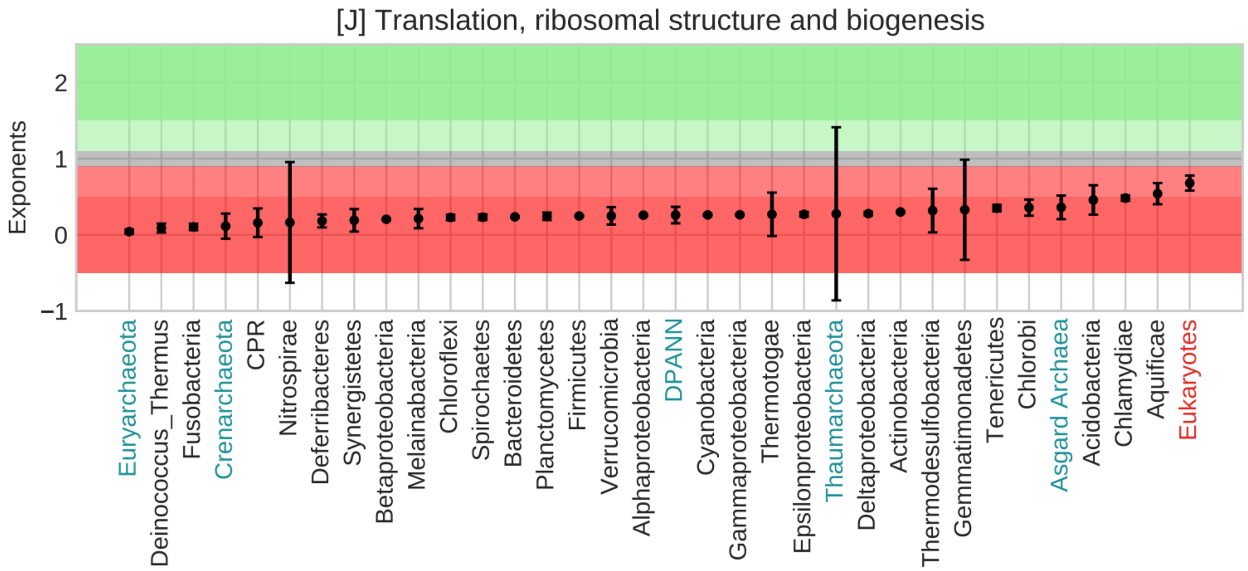

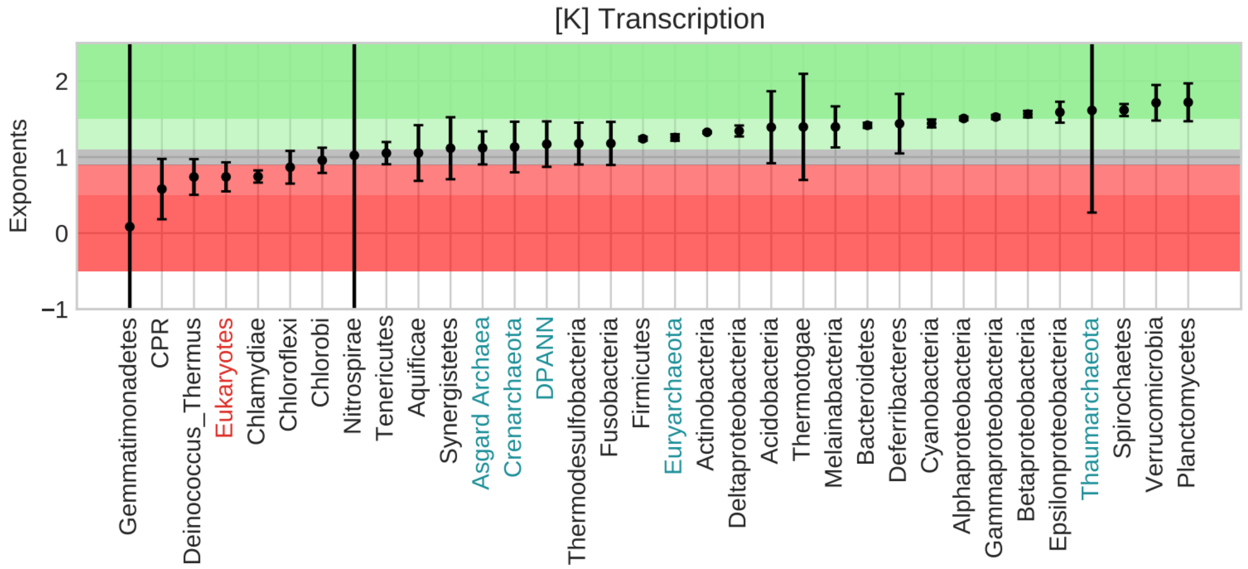

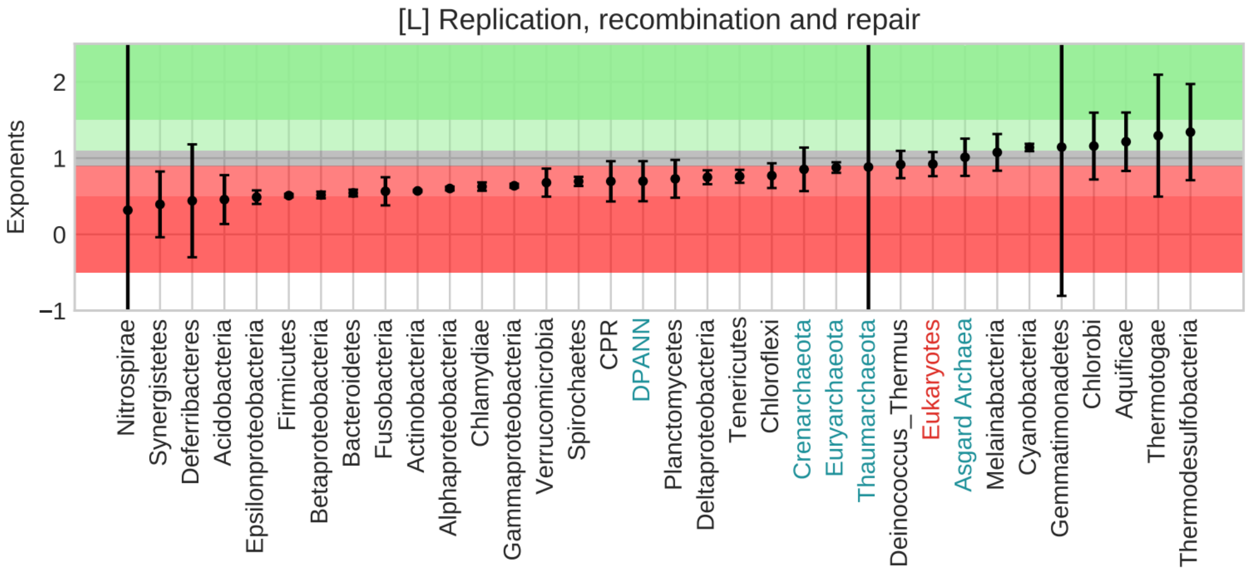

2] Cellular Processes and Signaling: COG categories D, M, N, O, T, U, V, W, Y, and Z

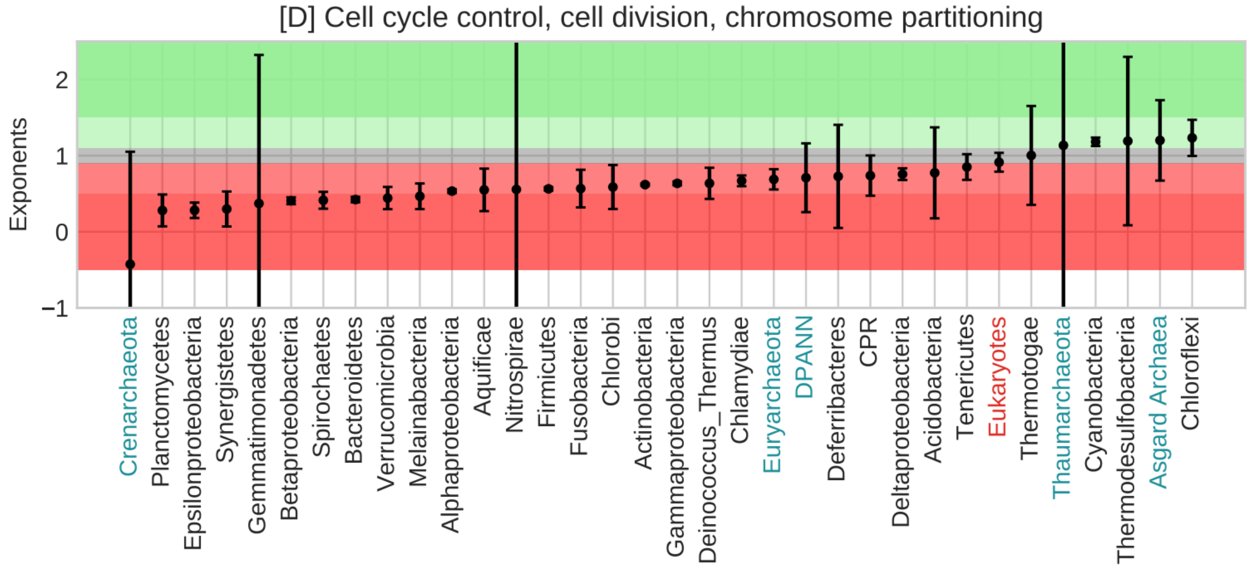

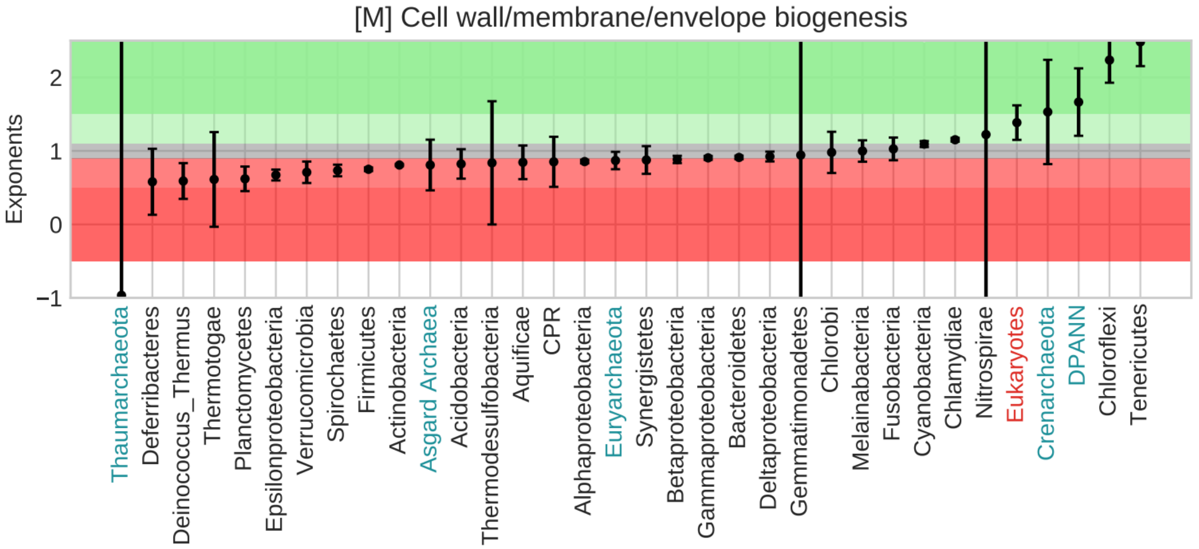
[M] Cell wall/membrane/envelope biogenesis
Exponents
2
1
0
−1
Thaumarchaeota
Deferribacteres
Deinococcus_Thermus
Thermotogae
Planctomycetes
Epsilonproteobacteria
Verrucomicrobia
Spirochaetes
Firmicutes
Actinobacteria
Asgard Archaea
Acidobacteria
Thermodesulfobacteria
Aquificae
CPR
Alphaproteobacteria
Euryarchaeota
Synergistetes
Betaproteobacteria
Gammaproteobacteria
Bacteroidetes
Deltaproteobacteria
Gemmatimonadetes
Chlorobi
Melainabacteria
Fusobacteria
Cyanobacteria
Chlamydiae
Nitrospirae
Eukaryotes
Crenarchaeota
DPANN
Chloroflexi
Tenericutes

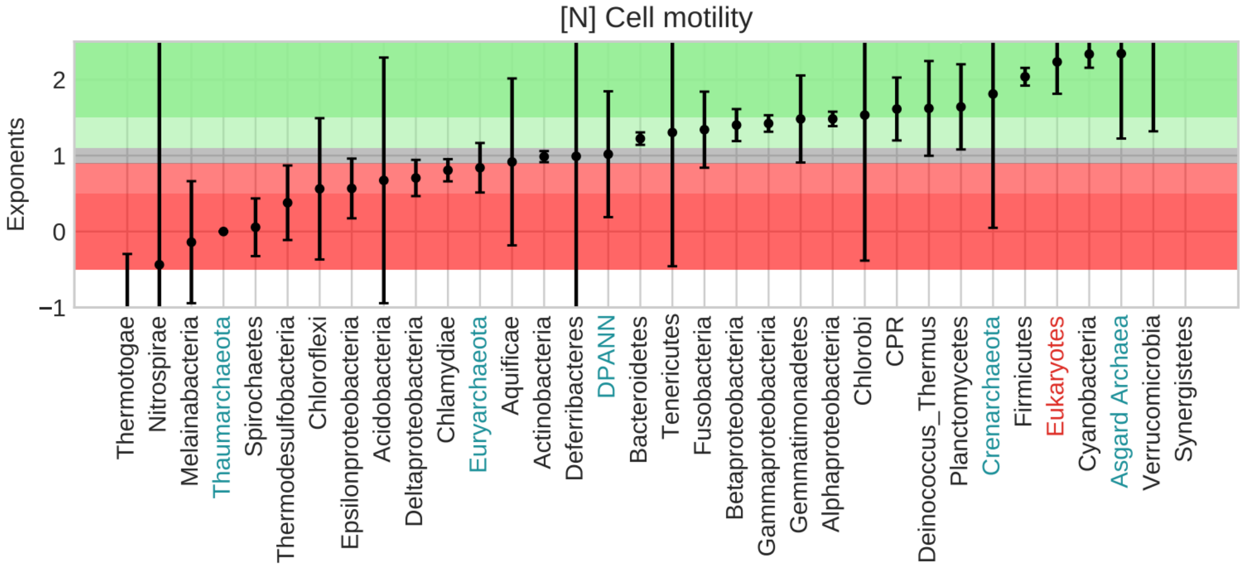
[N] Cell motility
Exponents
2
1
0
−1
Thermotogae
Nitrospirae
Melainabacteria
Thaumarchaeota
Spirochaetes
Thermodesulfobacteria
Chloroflexi
Epsilonproteobacteria
Acidobacteria
Deltaproteobacteria
Chlamydiae
Euryarchaeota
Aquificae
Actinobacteria
Deferribacteres
DPANN
Bacteroidetes
Tenericutes
Fusobacteria
Betaproteobacteria
Gammaproteobacteria
Gemmatimonadetes
Alphaproteobacteria
Chlorobi
CPR
Deinococcus_Thermus
Planctomycetes
Crenarchaeota
Firmicutes
Eukaryotes
Cyanobacteria
Asgard Archaea
Verrucomicrobia
Synergistetes

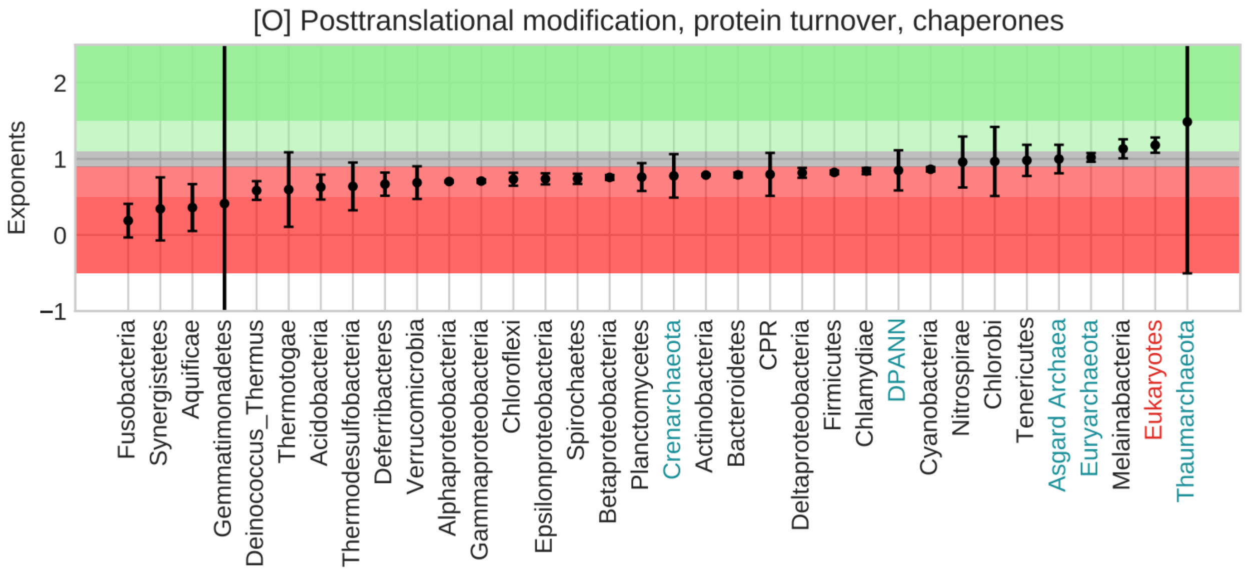
[O] Posttranslational modification, protein turnover, chaperones
Exponents
2
1
0
−1
Fusobacteria
Synergistetes
Aquificae
Gemmatimonadetes
Deinococcus_Thermus
Thermotogae
Acidobacteria
Thermodesulfobacteria
Deferribacteres
Verrucomicrobia
Alphaproteobacteria
Gammaproteobacteria
Chloroflexi
Epsilonproteobacteria
Spirochaetes
Betaproteobacteria
Planctomycetes
Crenarchaeota
Actinobacteria
Bacteroidetes
CPR
Deltaproteobacteria
Firmicutes
Chlamydiae
DPANN
Cyanobacteria
Nitrospirae
Chlorobi
Tenericutes
Asgard Archaea
Euryarchaeota
Melainabacteria
Eukaryotes
Thaumarchaeota

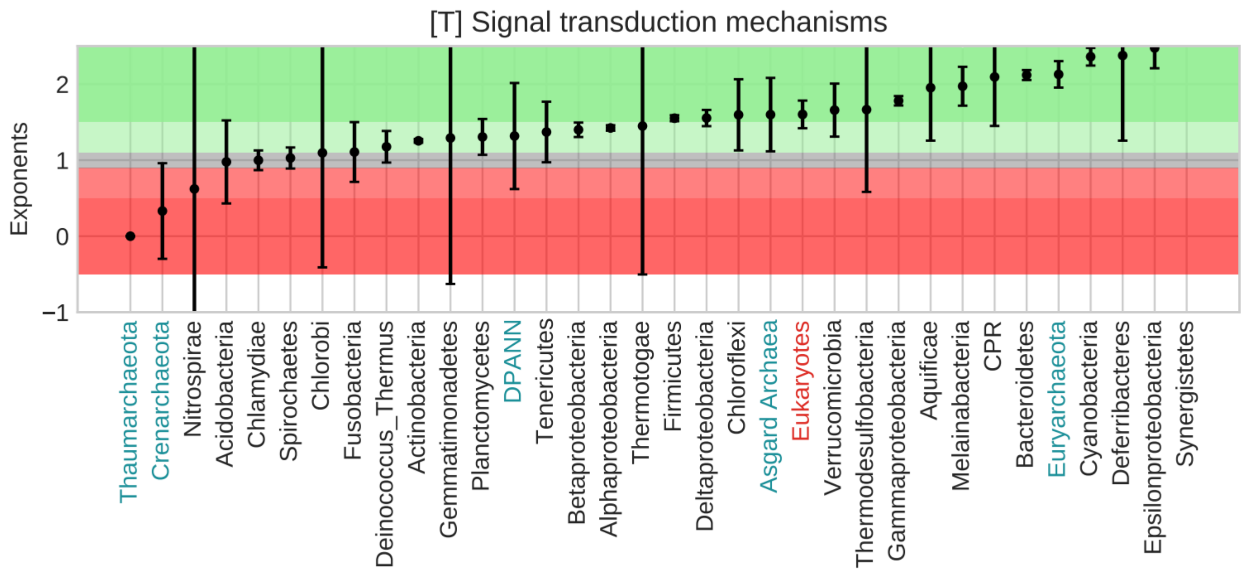
[T] Signal transduction mechanisms
Exponents
2
1
0
−1
Thaumarchaeota
Crenarchaeota
Nitrospirae
Acidobacteria
Chlamydiae
Spirochaetes
Chlorobi
Fusobacteria
Deinococcus_Thermus
Actinobacteria
Gemmatimonadetes
Planctomycetes
DPANN
Tenericutes
Betaproteobacteria
Alphaproteobacteria
Thermotogae
Firmicutes
Deltaproteobacteria
Chloroflexi
Asgard Archaea
Eukaryotes
Verrucomicrobia
Thermodesulfobacteria
Gammaproteobacteria
Aquificae
Melainabacteria
CPR
Bacteroidetes
Euryarchaeota
Cyanobacteria
Deferribacteres
Epsilonproteobacteria
Synergistetes

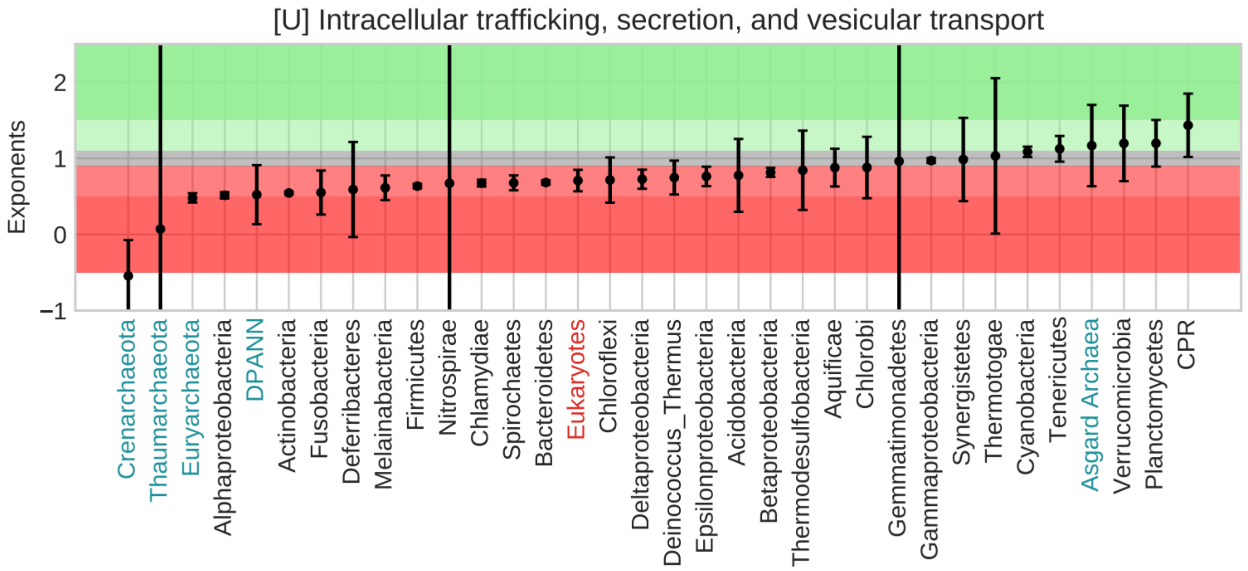
[U] Intracellular trafficking, secretion, and vesicular transport
Exponents
2
1
0
−1
Crenarchaeota
Thaumarchaeota
Euryarchaeota
Alphaproteobacteria
DPANN
Actinobacteria
Fusobacteria
Deferribacteres
Melainabacteria
Firmicutes
Nitrospirae
Chlamydiae
Spirochaetes
Bacteroidetes
Eukaryotes
Chloroflexi
Deltaproteobacteria
Deinococcus_Thermus
Epsilonproteobacteria
Acidobacteria
Betaproteobacteria
Thermodesulfobacteria
Aquificae
Chlorobi
Gemmatimonadetes
Gammaproteobacteria
Synergistetes
Thermotogae
Cyanobacteria
Tenericutes
Asgard Archaea
Verrucomicrobia
Planctomycetes
CPR

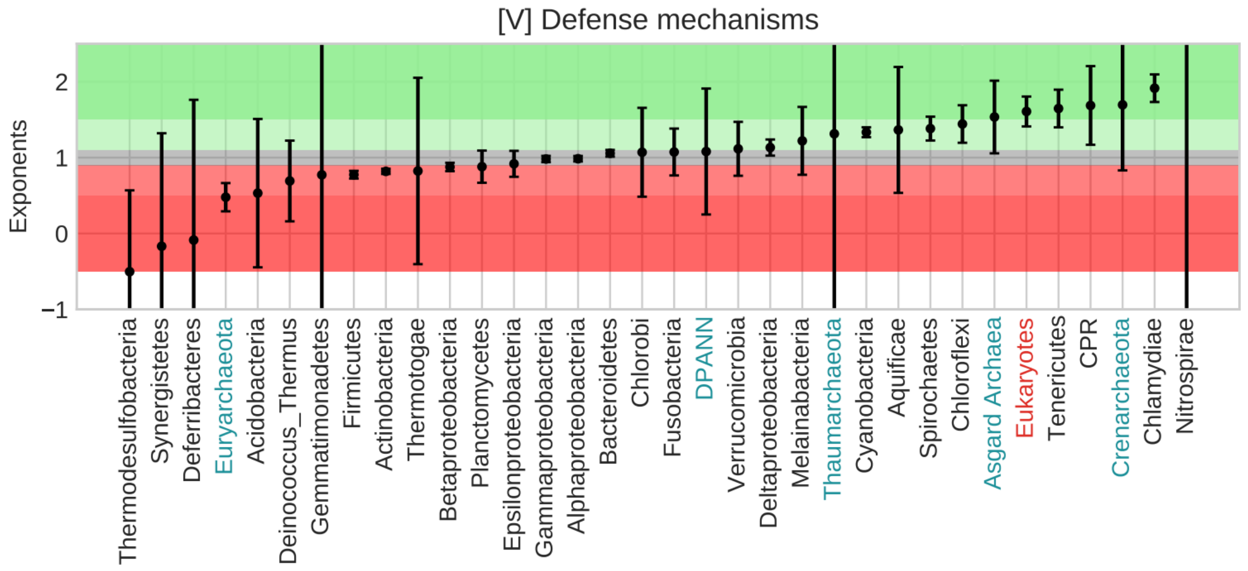
[V] Defense mechanisms
Exponents
2
1
0
−1
Thermodesulfobacteria
Synergistetes
Deferribacteres
Euryarchaeota
Acidobacteria
Deinococcus_Thermus
Gemmatimonadetes
Firmicutes
Actinobacteria
Thermotogae
Betaproteobacteria
Planctomycetes
Epsilonproteobacteria
Gammaproteobacteria
Alphaproteobacteria
Bacteroidetes
Chlorobi
Fusobacteria
DPANN
Verrucomicrobia
Deltaproteobacteria
Melainabacteria
Thaumarchaeota
Cyanobacteria
Aquificae
Spirochaetes
Chloroflexi
Asgard Archaea
Eukaryotes
Tenericutes
CPR
Crenarchaeota
Chlamydiae
Nitrospirae

3] Metabolism: COG Categories C, E, F, G, H, I, P, and Q

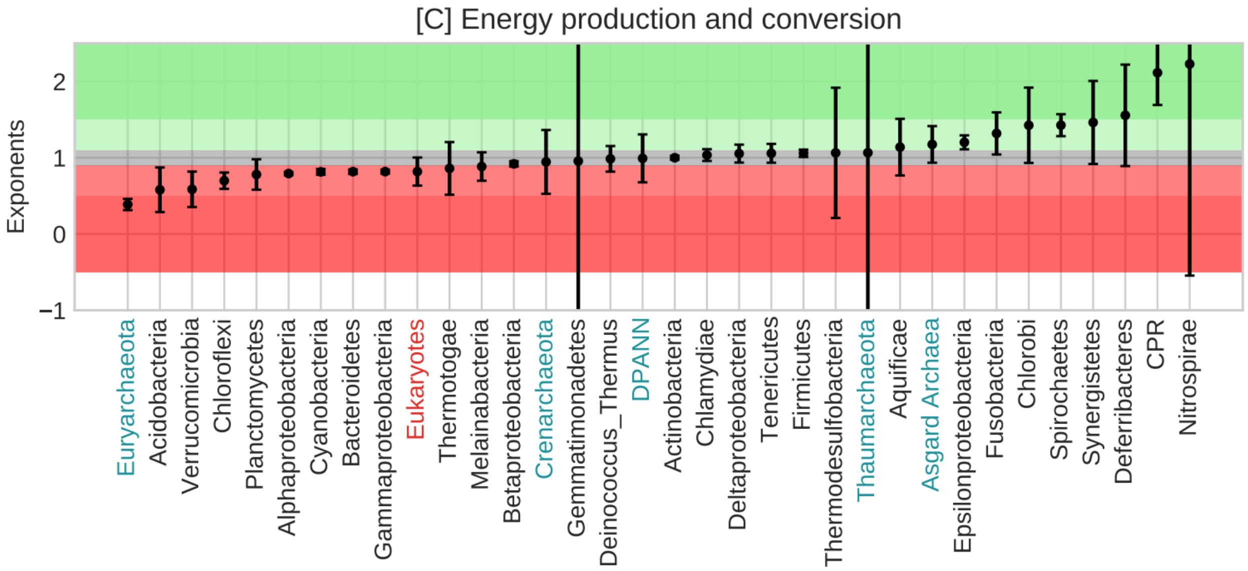

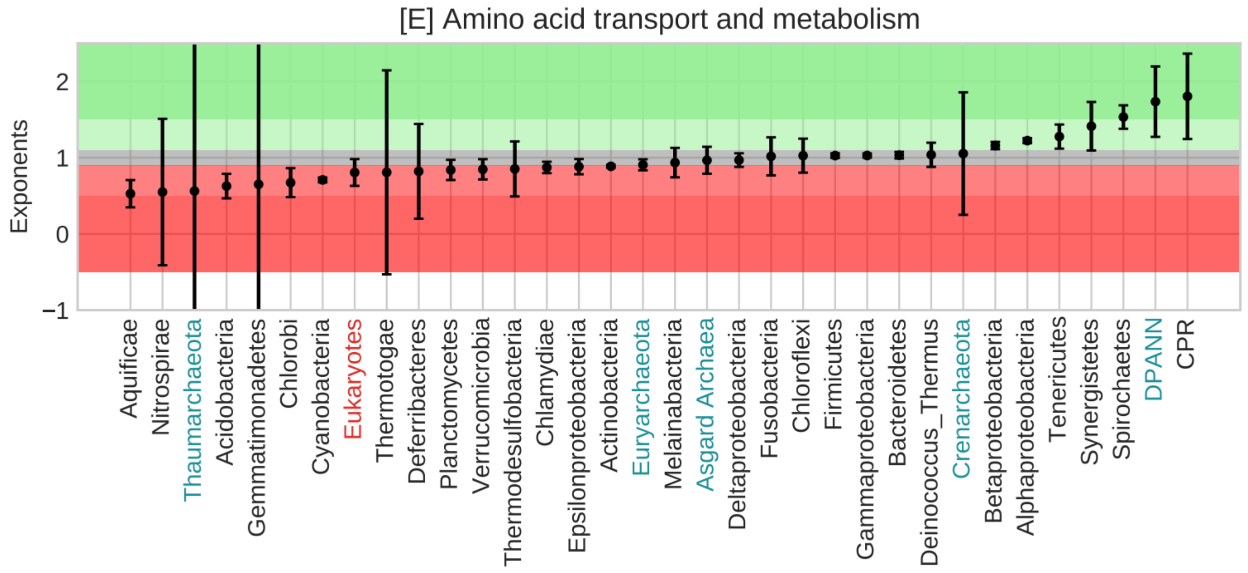

[F] Nucleotide transport and metabolism

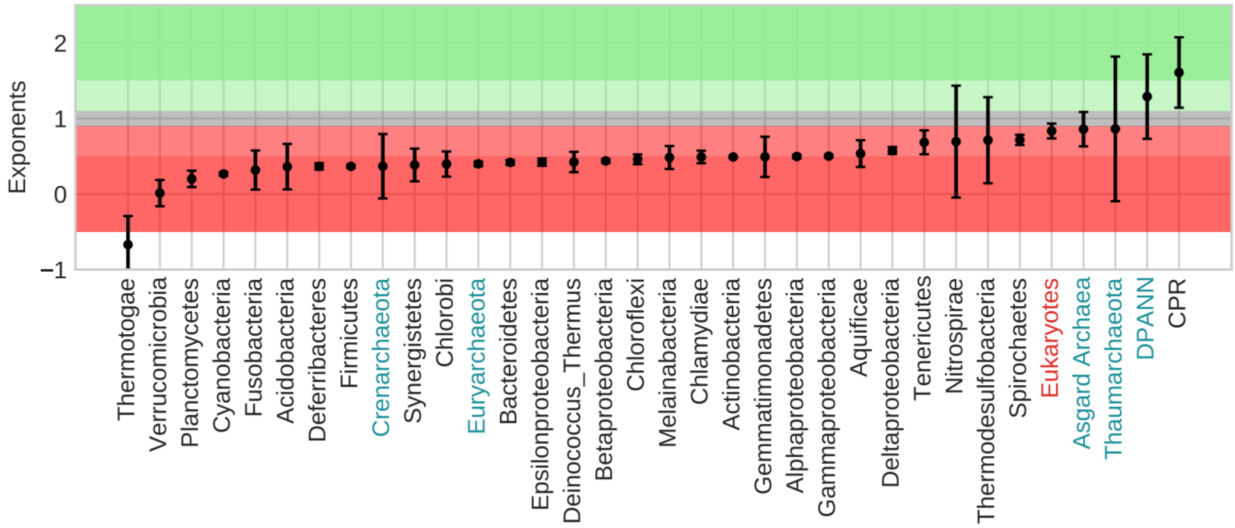

[G] Carbohydrate transport and metabolism

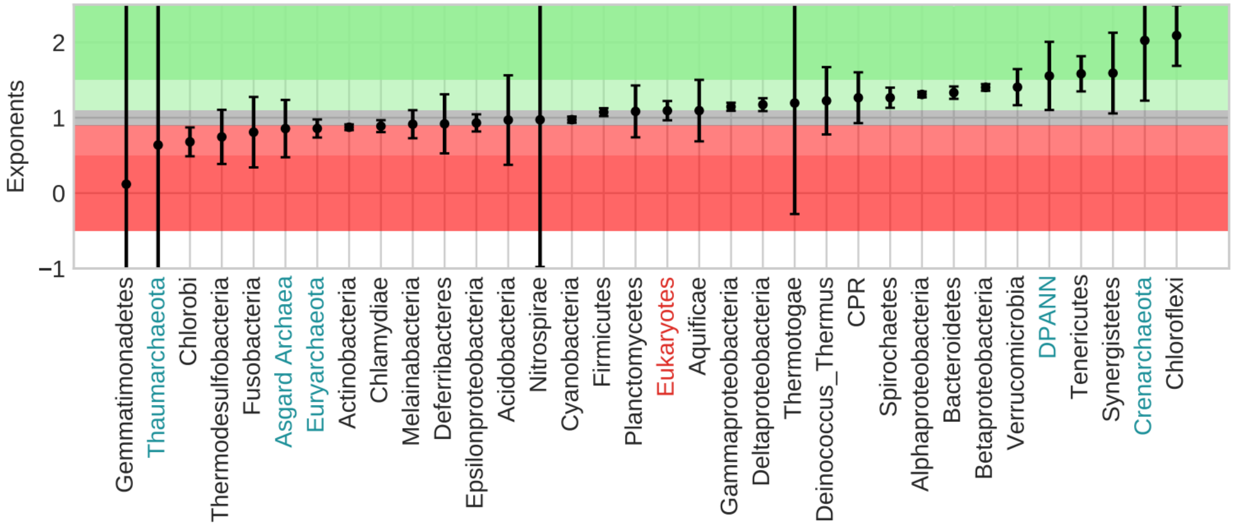

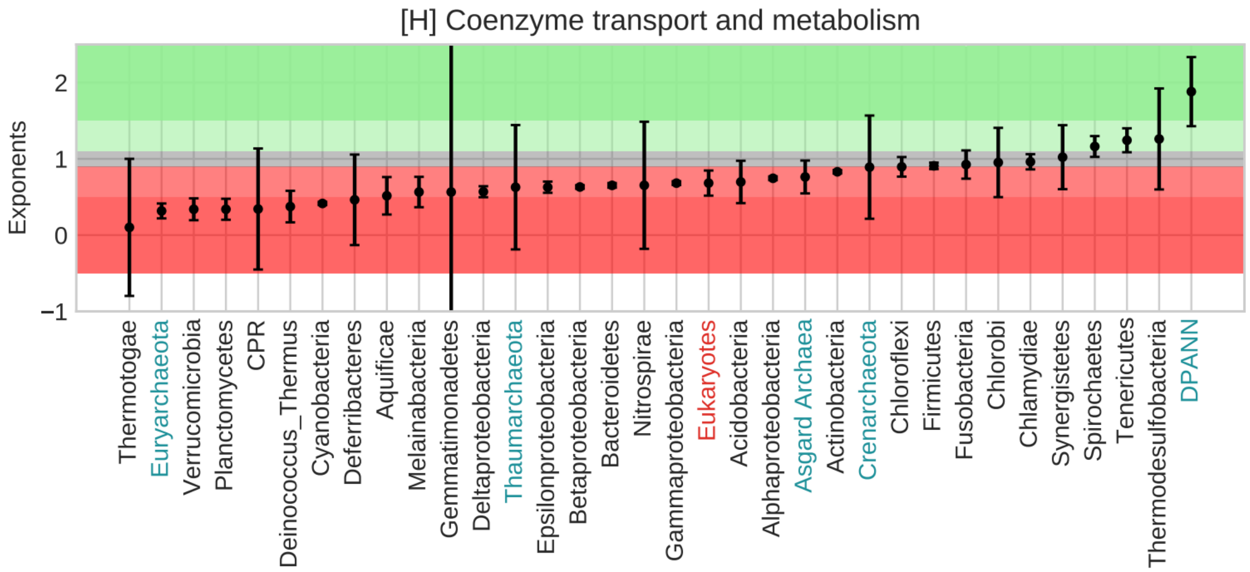
[H] Coenzyme transport and metabolism
Exponents
2
1
0
−1
Thermotogae
Euryarchaeota
Verrucomicrobia
Planctomycetes
CPR
Deinococcus_Thermus
Cyanobacteria
Deferribacteres
Aquificae
Melainabacteria
Gemmatimonadetes
Deltaproteobacteria
Thaumarchaeota
Epsilonproteobacteria
Betaproteobacteria
Bacteroidetes
Nitrospirae
Gammaproteobacteria
Eukaryotes
Acidobacteria
Alphaproteobacteria
Asgard Archaea
Actinobacteria
Crenarchaeota
Chloroflexi
Firmicutes
Fusobacteria
Chlorobi
Chlamydiae
Synergistetes
Spirochaetes
Tenericutes
Thermodesulfobacteria
DPANN

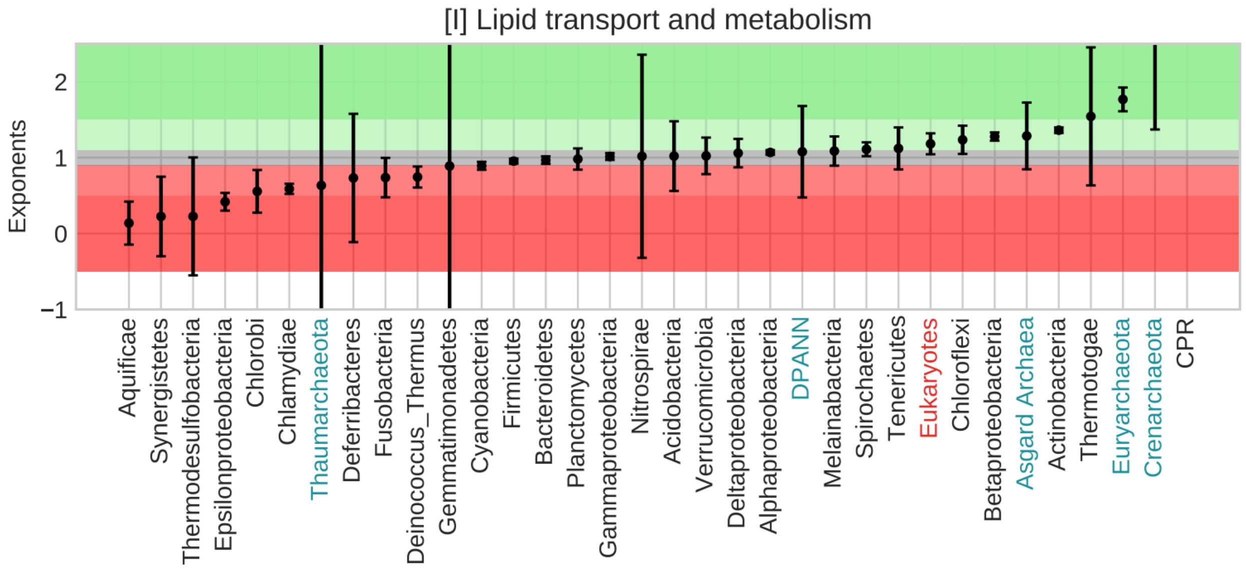
[I] Lipid transport and metabolism
Exponents
2
1
0
−1
Aquificae
Synergistetes
Thermodesulfobacteria
Epsilonproteobacteria
Chlorobi
Chlamydiae
Thaumarchaeota
Deferribacteres
Fusobacteria
Deinococcus_Thermus
Gemmatimonadetes
Cyanobacteria
Firmicutes
Bacteroidetes
Planctomycetes
Gammaproteobacteria
Nitrospirae
Acidobacteria
Verrucomicrobia
Deltaproteobacteria
Alphaproteobacteria
DPANN
Melainabacteria
Spirochaetes
Tenericutes
Eukaryotes
Chloroflexi
Betaproteobacteria
Asgard Archaea
Actinobacteria
Thermotogae
Euryarchaeota
Crenarchaeota
CPR

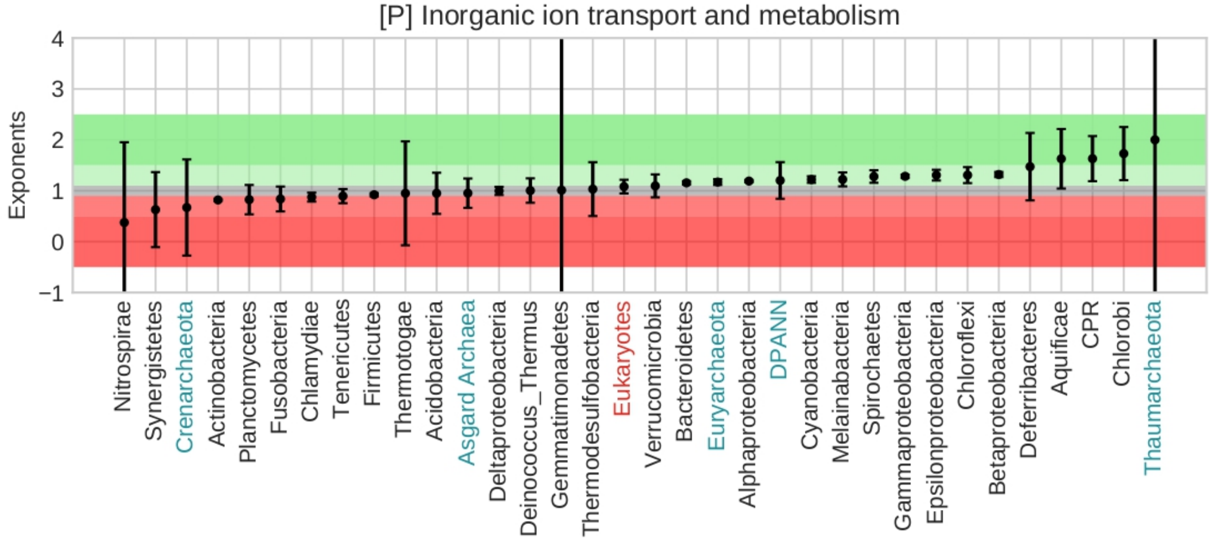
[P] Inorganic ion transport and metabolism
Exponents
4
3
2
1
0
−1
Nitrospirae
Synergistetes
Crenarchaeota
Actinobacteria
Planctomycetes
Fusobacteria
Chlamydiae
Tenericutes
Firmicutes
Thermotogae
Acidobacteria
Asgard Archaea
Deltaproteobacteria
Deinococcus_Thermus
Gemmatimonadetes
Thermodesulfobacteria
Eukaryotes
Verrucomicrobia
Bacteroidetes
Euryarchaeota
Alphaproteobacteria
DPANN
Cyanobacteria
Melainabacteria
Spirochaetes
Gammaproteobacteria
Epsilonproteobacteria
Chloroflexi
Betaproteobacteria
Deferribacteres
Aquificae
CPR
Chlorobi
Thaumarchaeota

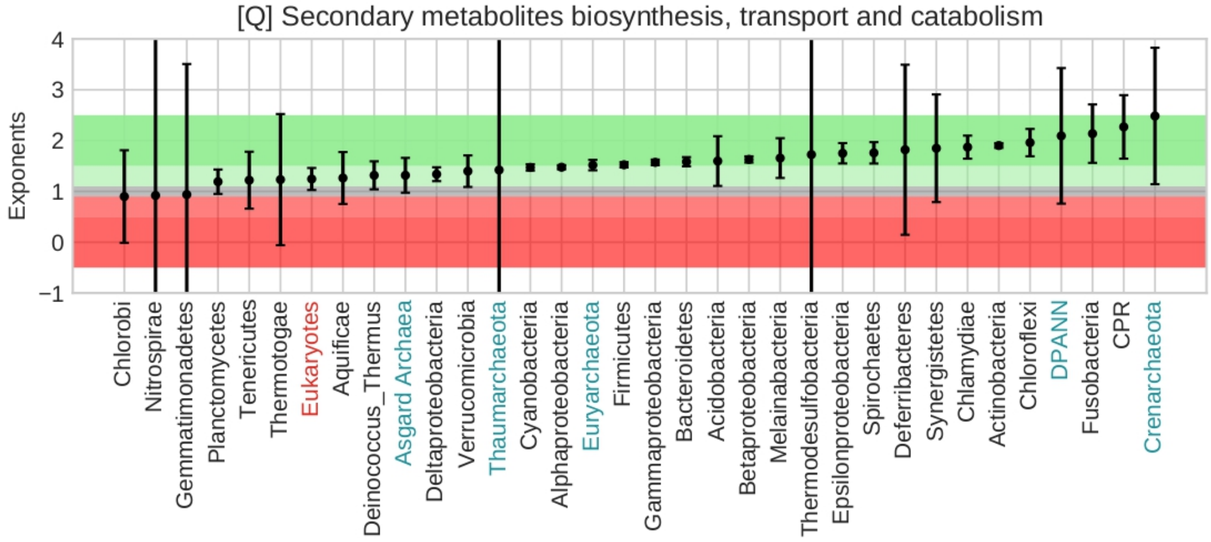
[Q] Secondary metabolites biosynthesis, transport and catabolism
Exponents
4
3
2
1
0
−1
Chlorobi
Nitrospirae
Gemmatimonadetes
Planctomycetes
Tenericutes
Thermotogae
Eukaryotes
Aquificae
Deinococcus_Thermus
Asgard Archaea
Deltaproteobacteria
Verrucomicrobia
Thaumarchaeota
Cyanobacteria
Alphaproteobacteria
Euryarchaeota
Firmicutes
Gammaproteobacteria
Bacteroidetes
Acidobacteria
Betaproteobacteria
Melainabacteria
Thermodesulfobacteria
Epsilonproteobacteria
Spirochaetes
Deferribacteres
Synergistetes
Chlamydiae
Actinobacteria
Chloroflexi
DPANN
Fusobacteria
CPR
Crenarchaeota

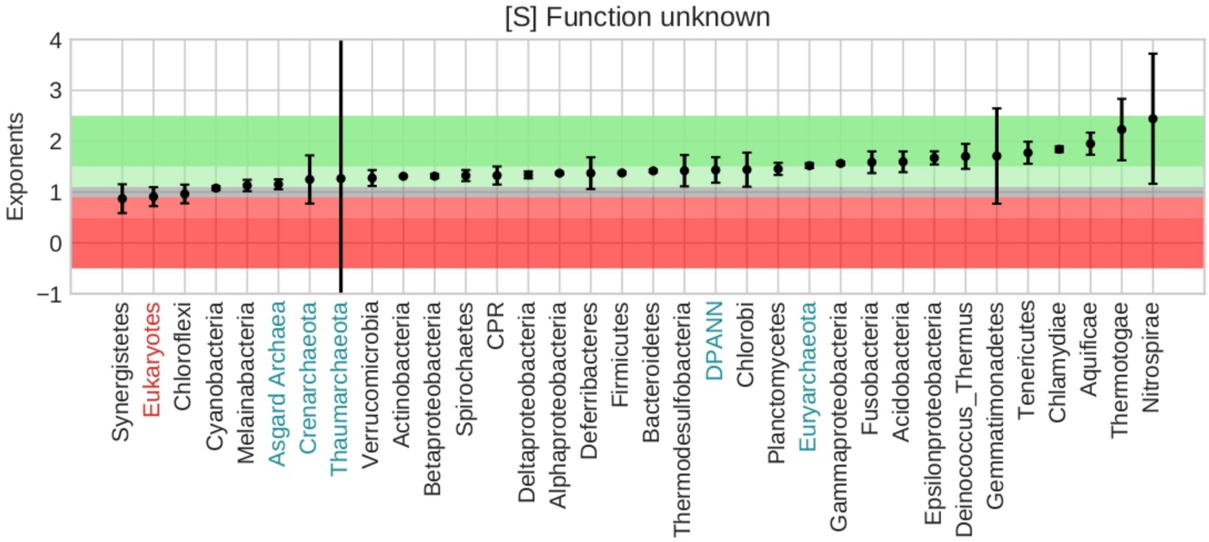

Supplementary Figure 4: Phyla arranged in the increasing order of their exponents with 95% confidence intervals. Background horizontal span colors signify scaling. From dark green to dark red, meaning superlinear scaling to sublinear scaling. The grey span in between signifies linear scaling.
X-axis labels: Bacterial phyla in black, archaeal phyla in cyan, and Eukaryotes in red.

1] Information Storage and Processing: COG categories A, B, J, K, and L

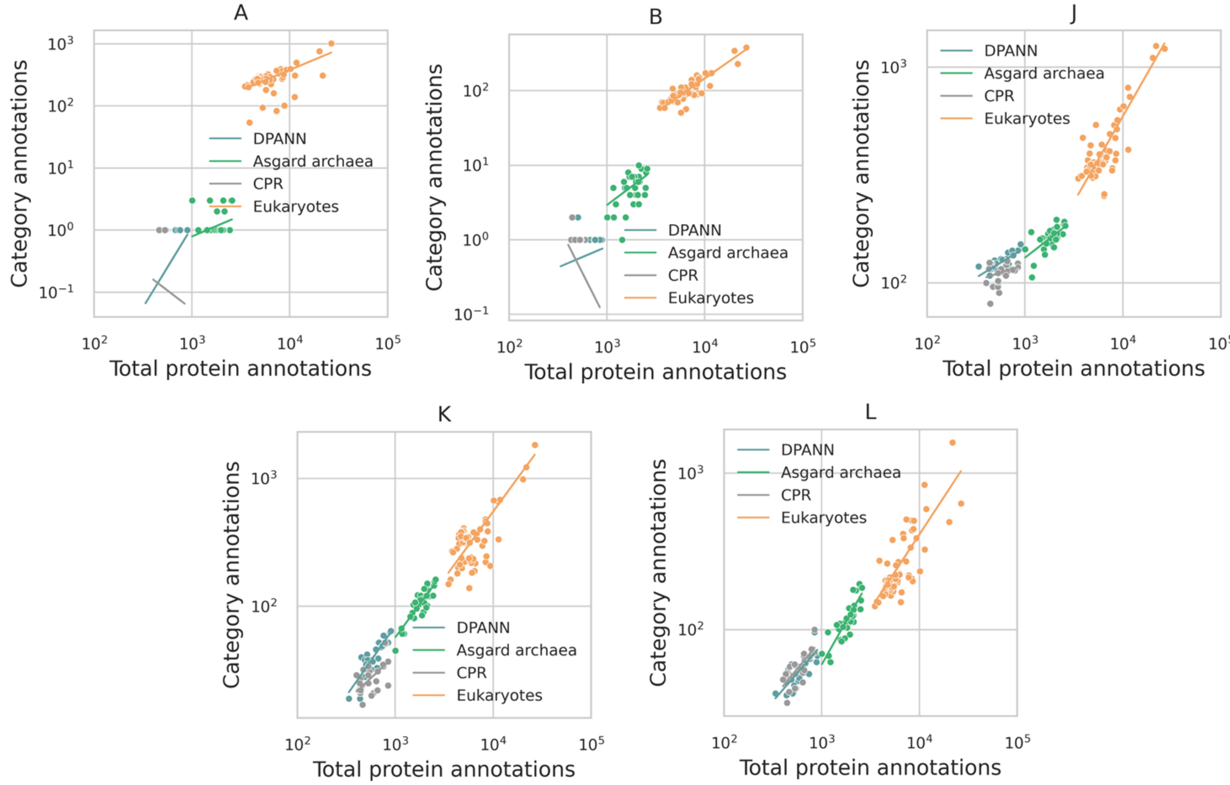

2] Cellular Processes and Signaling: COG categories D, M, N, O, T, U, V, W, Y, and Z

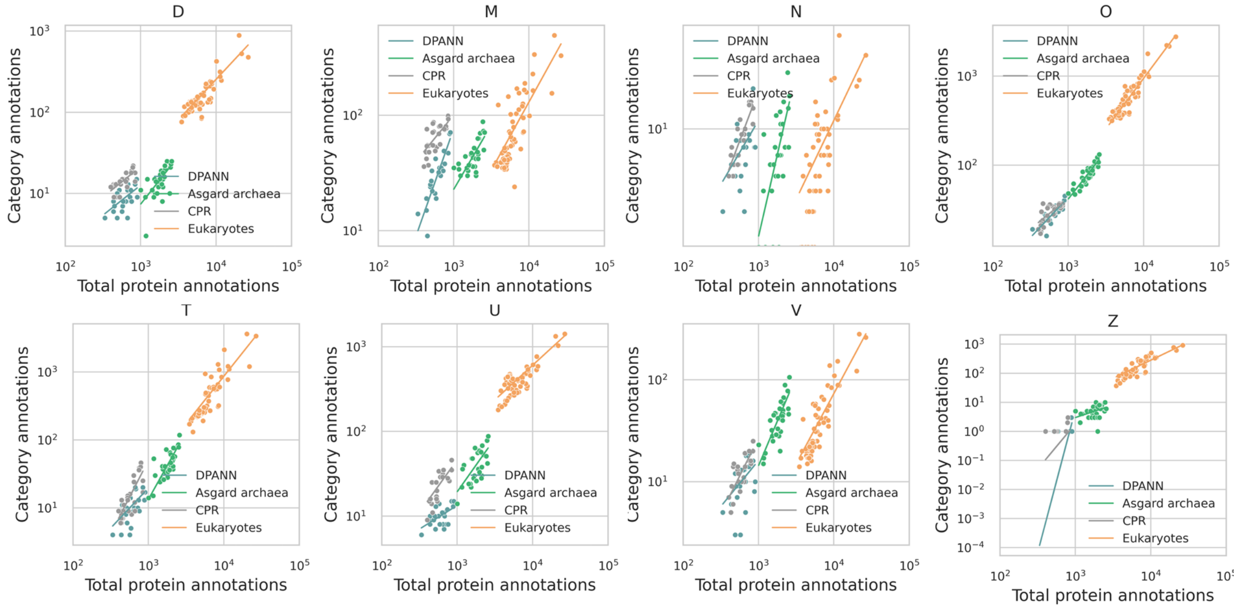

3] Metabolism: COG Categories C, E, F, G, H, I, P, and Q

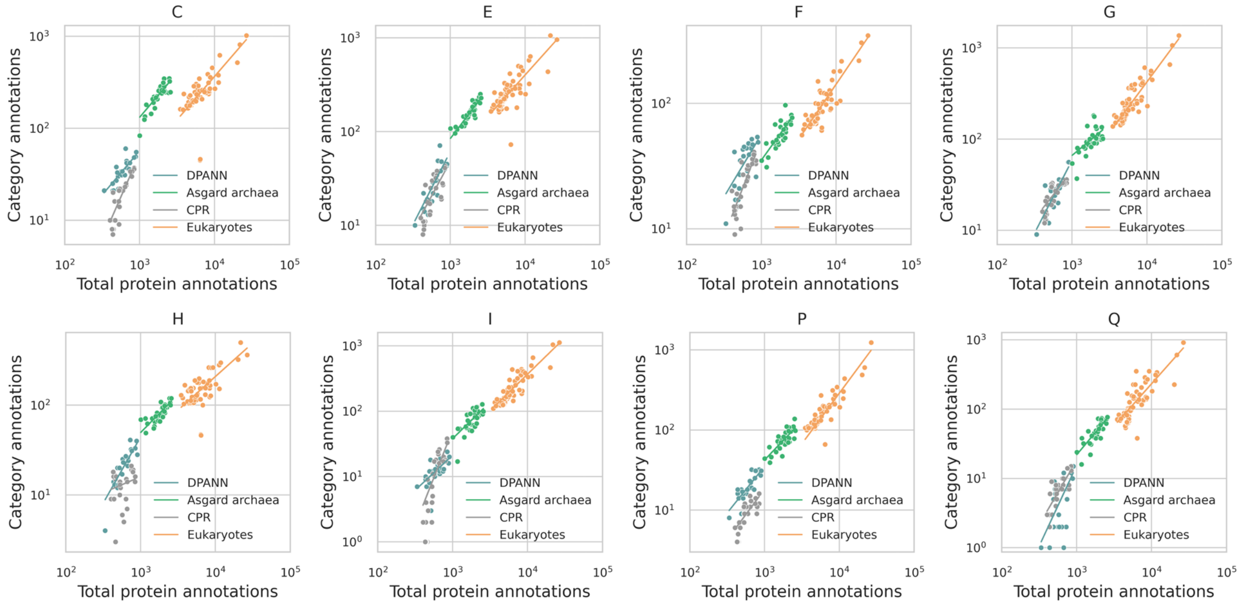

Supplementary Figure 5: Scaling comparison between CPR, DPANN, Asgard archaea, and Eukaryotes
